# Supplementary material for: Topographical archetypes of somatic mutagenesis in cancer
Source: bioRxiv. 2026 Apr 21:2026.04.18.719374. Preprint. [Version 1] doi: 10.64898/2026.04.18.719374 (PMC13131822; doi:10.64898/2026.04.18.719374)
Supplement: 1 [file NIHPP2026.04.18.719374V1-supplement-1.pdf]

bioRxiv preprint doi: https://doi.org/10.64898/2026.04.18.719374; this version posted April 21, 2026. The copyright holder for this preprint (which was not certified by peer review) is the author/funder, who has granted bioRxiv a license to display the preprint in perpetuity. It is made available under aCC-BY 4.0 International license.

Supplementary methods

Overview of the probabilistic model. MuTopia is a latent-variable generative model for somatic mutations across genomic bins and mutation types. For each sample n, the model introduces a vector of normalized process exposures $\pi_n \in \Delta_{K-1}$, drawn from a Dirichlet prior, $\pi_n \sim \mathrm{Dir}(\alpha)$. Each mutation is then generated in two steps: first, a latent mutational process $z_{ni} \in \{1, \ldots, K\}$ is sampled from a categorical distribution with parameters π_n, and second, a genomic bin b_{ni} and mutation type m_{ni} are sampled from the process-specific distribution $p(b, m \mid z_{ni} = k, \theta)$. The parameters θ define these process-specific topographies through the macro-scale functions f_k and spectra functions g_k, together with the context-availability term t_{bm}. In this way, MuTopia combines sample-specific mixture proportions with process-specific, feature-dependent mutation distributions across the genome.

Mutation-level likelihood. Let M_n denote the number of mutations observed in sample n (the mutational burden). We write

$$z_n = (z_{n1}, \ldots, z_{nM_n}), \qquad b_n = (b_{n1}, \ldots, b_{nM_n}), \qquad m_n = (m_{n1}, \ldots, m_{nM_n})$$

for the vectors of latent process assignments, genomic bins, and mutation types in sample n. Under the model, we have $p(z_{ni} = k \mid \pi_n) = \pi_{nk}$, and the complete-data likelihood for sample n therefore factorizes as

$$p(b_n, m_n, z_n, \pi_n \mid \alpha, \theta) = p(\pi_n \mid \alpha) \prod_i \pi_{n z_{ni}} \, p(b_{ni}, m_{ni} \mid z_{ni}, \theta).$$

Marginalizing over the latent assignments z_n gives

$$p(b_n, m_n, \pi_n \mid \alpha, \theta) = p(\pi_n \mid \alpha) \prod_i \sum_k \pi_{nk} \, p(b_{ni}, m_{ni} \mid z_{ni} = k, \theta).$$

Finally, marginalizing over the latent exposures and taking the product over samples yields the marginal likelihood

$$\prod_n \int p(\pi_n \mid \alpha) \prod_i \sum_k \pi_{nk} \, p(b_{ni}, m_{ni} \mid z_{ni} = k, \theta) \, d\pi_n.$$

Count-level likelihood. Now let

$$Y_{nbm} = \sum_i \mathbf{1}_{(b_{ni}, m_{ni}) = (b, m)}$$

denote the number of mutations of type m observed in bin b for sample n. Since the mutation-level likelihood depends on the observations only through the counts of each bin–type pair in each sample, we can group identical terms to rewrite the marginal likelihood as

$$p(Y \mid \alpha, \theta) = \prod_n \int p(\pi_n \mid \alpha) \prod_{b,m} \left(\sum_k \pi_{nk} \, p(b, m \mid z = k, \theta) \right)^{Y_{nbm}} d\pi_n.$$

This is the same marginal likelihood as above, rewritten in terms of the aggregated counts Y_{nbm}.

Variational inference. Direct maximization of the marginal likelihood is intractable because it requires integrating over the latent exposures and summing over all mutation-to-process assignments. Variational inference addresses this by introducing a tractable family of distributions over the latent variables and deriving the evidence lower bound (ELBO), a lower bound on the marginal log-likelihood that can be computed efficiently. Maximizing the ELBO yields a practical surrogate objective for learning the model parameters (α, θ) while simultaneously approximating the posterior distribution over the latent variables [1].

bioRxiv preprint doi: https://doi.org/10.64898/2026.04.18.719374; this version posted April 21, 2026. The copyright holder for this preprint (which was not certified by peer review) is the author/funder, who has granted bioRxiv a license to display the preprint in perpetuity. It is made available under aCC-BY 4.0 International license.

We begin with a mean-field variational approximation at the mutation level,

$$q(\pi, z) = \prod_n q(\pi_n \mid \beta_n) \prod_i q(z_{ni} \mid \varphi_{ni}),$$

where $q(\pi_n \mid \beta_n)$ is Dirichlet and $q(z_{ni} \mid \varphi_{ni})$ is categorical. For a single sample n, the ELBO is

$$\mathcal{L}_n(\beta_n, \varphi_n, \alpha, \theta) = \mathbb{E}_{q(\pi_n, z_n)}\big[\log p(b_n, m_n, z_n, \pi_n \mid \alpha, \theta)\big] - \mathbb{E}_{q(\pi_n, z_n)}\big[\log q(\pi_n, z_n)\big].$$

Summing over samples gives the full ELBO

$$\mathcal{L}(\beta, \varphi, \alpha, \theta) = \sum_n \mathcal{L}_n(\beta_n, \varphi_n, \alpha, \theta). \tag{1}$$

Mutation-level ELBO. The explicit derivation of the ELBO is identical to that of latent Dirichlet allocation (LDA) [2], except that the vocabulary index is replaced here by the genomic bin–mutation-type pair (b, m). Using the mean-field variational family above, the per-sample ELBO can be written as

$$\mathcal{L}_n = \mathbb{E}_{q(\pi_n)}[\log p(\pi_n \mid \alpha)] + \mathbb{E}_{q(\pi_n, z_n)}[\log p(b_n, m_n, z_n \mid \pi_n, \theta)] - \mathbb{E}_{q(\pi_n)}[\log q(\pi_n)] - \mathbb{E}_{q(z_n)}[\log q(z_n)].$$

Since $q(\pi_n \mid \beta_n)$ is Dirichlet, the prior term is

$$\mathbb{E}_{q(\pi_n)}[\log p(\pi_n \mid \alpha)] = \log\Gamma\big(\textstyle\sum_k \alpha_k\big) - \displaystyle\sum_k \log\Gamma(\alpha_k) + \sum_k (\alpha_k - 1)\, \mathbb{E}_{q(\pi_n)}[\log \pi_{nk}],$$

and the variational entropy term is

$$\mathbb{E}_{q(\pi_n)}[\log q(\pi_n)] = \log\Gamma\big(\textstyle\sum_k \beta_{nk}\big) - \displaystyle\sum_k \log\Gamma(\beta_{nk}) + \sum_k (\beta_{nk} - 1)\, \mathbb{E}_{q(\pi_n)}[\log \pi_{nk}].$$

Here, Γ denotes the Gamma function, and the expectation can be computed explicitly using the digamma function ψ via

$$\mathbb{E}_{q(\pi_n)}[\log \pi_{nk}] = \psi(\beta_{nk}) - \psi\big(\textstyle\sum_{k'} \beta_{nk'}\big). \tag{2}$$

Next, we have

$$\mathbb{E}_{q(\pi_n, z_n)}[\log p(b_n, m_n, z_n \mid \pi_n, \theta)] = \sum_{i,k} \varphi_{nik}\big(\mathbb{E}_{q(\pi_n)}[\log \pi_{nk}] + \log p(b_{ni}, m_{ni} \mid z = k, \theta)\big)$$

and

$$\mathbb{E}_{q(z_n)}[\log q(z_n)] = \sum_{i,k} \varphi_{nik} \log \varphi_{nik}.$$

Combining these expressions and using

$$\widetilde{\pi}_{nk} := \exp\big(\mathbb{E}_{q(\pi_n)}[\log \pi_{nk}]\big) \tag{3}$$

for notational convenience gives

$$\begin{aligned}\mathcal{L}_n(\beta_n, \varphi_n, \alpha, \theta) = \sum_{i,k} \varphi_{nik} \log \frac{p(b_{ni}, m_{ni} \mid z = k, \theta)\, \widetilde{\pi}_{nk}}{\varphi_{nik}} + \log\Gamma\big(\textstyle\sum_k \alpha_k\big) - \log\Gamma\big(\textstyle\sum_k \beta_{nk}\big) \\ + \sum_k \Big(\log\Gamma(\beta_{nk}) - \log\Gamma(\alpha_k) + (\alpha_k - \beta_{nk}) \log \widetilde{\pi}_{nk}\Big).\end{aligned} \tag{4}$$

Summing over samples gives the full ELBO.

bioRxiv preprint doi: https://doi.org/10.64898/2026.04.18.719374; this version posted April 21, 2026. The copyright holder for this preprint (which was not certified by peer review) is the author/funder, who has granted bioRxiv a license to display the preprint in perpetuity. It is made available under aCC-BY 4.0 International license.

Count-level ELBO. Because the likelihood depends on each mutation only through its observed bin–type pair (b_{ni}, m_{ni}), mutations in the same sample with the same pair (b, m) are exchangeable under both the model and the variational family. Consequently, the optimal variational parameters satisfy

$$\varphi_{ni} = \phi_{nbm} \qquad \text{whenever } (b_{ni}, m_{ni}) = (b, m),$$

for some shared $\phi_{nbm} \in \Delta_{K-1}$. Thus, although the variational family was introduced at the mutation level, the optimal variational distribution can be reparameterized using one probabilistic mutation-to-process assignment per sample, bin, and mutation type. Grouping the terms in (4) by (b, m) then yields

$$\begin{aligned}\mathcal{L}_n(\beta_n, \phi_n, \alpha, \theta) = &\sum_{b,m,k} Y_{nbm}\phi_{nbmk} \log \frac{p(b, m \mid z = k, \theta)\, \widetilde{\pi}_{nk}}{\phi_{nbmk}} + \log \Gamma\big(\textstyle\sum_k \alpha_k\big) - \log \Gamma\big(\textstyle\sum_k \beta_{nk}\big) \\ &+ \sum_k \Big(\log \Gamma(\beta_{nk}) - \log \Gamma(\alpha_k) + (\alpha_k - \beta_{nk}) \log \widetilde{\pi}_{nk} \Big).\end{aligned} \tag{5}$$

Summing over samples gives the full ELBO,

$$\mathcal{L}(\beta, \phi, \alpha, \theta) = \sum_n \mathcal{L}_n(\beta_n, \phi_n, \alpha, \theta). \tag{6}$$

This is the same ELBO as above, rewritten in terms of the aggregated counts Y_{nbm}. In practice, this representation is preferable because it avoids redundant variational parameters for mutations with identical observed bin–type pairs. We henceforth use the ϕ-parameterization of the model.

Model training

MuTopia model training proceeds by maximizing (6) via coordinate ascent: in the variational E-step, we update β and ϕ given the current model parameters, and in the M-step, we update α and θ given the current variational parameters. Intuitively, the E-step updates the approximate posterior over the sample-specific exposures and mutation-to-process assignments under the current model, and the M-step updates the process topographies and prior parameters using those variational estimates.

Optimizing the variational parameters

For fixed model parameters (α, θ), the variational E-step maximizes the ELBO with respect to the variational parameters ϕ and β. These updates are similar to LDA [2], except that the vocabulary index is replaced here by the genomic bin–mutation-type pair (b, m).

Updating ϕ_{nbmk}. The terms in $\mathcal{L}$ that depend on $\phi_{nbm} \in \Delta_{K-1}$ are

$$\sum_k Y_{nbm}\phi_{nbmk} \log \frac{p(b, m \mid z = k, \theta)\, \widetilde{\pi}_{nk}}{\phi_{nbmk}}.$$

Using a Lagrange multiplier for the simplex constraint, the optimum satisfies

$$\phi_{nbmk} \propto p(b, m \mid z = k, \theta)\, \widetilde{\pi}_{nk} \tag{7}$$

with normalization over k.

Updating β_{nk}. The terms in $\mathcal{L}$ that depend on β_{nk} are

$$\sum_{k'} \big(\alpha_{k'} + \textstyle\sum_{b,m} Y_{nbm}\phi_{nbmk'} - \beta_{nk'}\big) \log \widetilde{\pi}_{nk'} - \log \Gamma\big(\textstyle\sum_{k'} \beta_{nk'}\big) + \log \Gamma(\beta_{nk}).$$

bioRxiv preprint doi: https://doi.org/10.64898/2026.04.18.719374; this version posted April 21, 2026. The copyright holder for this preprint (which was not certified by peer review) is the author/funder, who has granted bioRxiv a license to display the preprint in perpetuity. It is made available under aCC-BY 4.0 International license.

Optimizing over β_n yields the maximum at

$$\beta_{nk} = \alpha_k + \sum_{b,m} Y_{nbm}\phi_{nbmk}. \tag{8}$$

Thus, β_{nk} is given by the prior pseudo-count α_k plus the expected number of mutations in sample n assigned to process k.

The variational E-step therefore consists of alternating the updates in (7) and (8) until convergence.

Optimizing the model parameters

Updating α. The terms in $\mathcal{L}$ that depend on α_k are

$$N_{\text{samples}}\Big(\log\Gamma\big(\textstyle\sum_{k'}\alpha_{k'}\big) - \log\Gamma(\alpha_k)\Big) + \alpha_k \sum_n \log\widetilde{\pi}_{nk}. \tag{9}$$

To optimize α, we follow [2, A.4.2] and use a Newton–Raphson algorithm with derivatives

$$\frac{\partial\mathcal{L}}{\partial\alpha_k} = N_{\text{samples}}\Big(\psi\big(\textstyle\sum_{k'}\alpha_{k'}\big) - \psi(\alpha_k)\Big) + \sum_n \log\widetilde{\pi}_{nk}$$

and Hessian

$$\frac{\partial^2\mathcal{L}}{\partial\alpha_k\alpha_{k'}} = N_{\text{samples}}\Big(\psi'\big(\textstyle\sum_{k''}\alpha_{k''}\big) - \delta_{kk'}\psi'(\alpha_k)\Big).$$

Updating θ. The terms in $\mathcal{L}$ that depend on θ are

$$\sum_{n,b,m,k} Y_{nbm}\phi_{nbmk}\log p(b,m \mid z=k,\theta).$$

Under any natural parameterization of the model, the topography parameters are process-specific rather than shared across mutational processes. Accordingly, we write $\theta = (\theta_1,\ldots,\theta_K)$, where θ_k denotes the parameters governing the topography of process k. Optimizing $\mathcal{L}$ with respect to θ is therefore equivalent to optimizing $\mathcal{L}$ with respect to each θ_k separately. For fixed k, we define the signature-assigned "soft counts"

$$\widetilde{Y}_{bm} := \sum_n Y_{nbm}\phi_{nbmk}. \tag{10}$$

The terms that depend on θ_k can then be written as

$$\sum_{b,m} \widetilde{Y}_{bm}\log p(b,m \mid z=k,\theta_k).$$

Thus, for each process k, the M-step reduces to maximizing a multinomial log-likelihood in which $\widetilde{Y}_{bm}$ acts as the expected number of mutations assigned to process k in bin b and mutation type m.

For any unnormalized mutation rate $\mu_{bm} = \mu_{bm}(\theta_k) > 0$, write the process-specific topography in the generic form

$$p(b,m \mid z=k,\theta_k) = \frac{\mu_{bm}}{\sum_{b',m'}\mu_{b'm'}}.$$

Substituting this into the objective gives

$$\sum_{b,m}\widetilde{Y}_{bm}\log p(b,m \mid z=k,\theta_k) = \sum_{b,m}\widetilde{Y}_{bm}\log\mu_{bm} - \Big(\sum_{b,m}\widetilde{Y}_{bm}\Big)\log\Big(\sum_{b,m}\mu_{bm}\Big). \tag{11}$$

Direct optimization of this multinomial objective is inconvenient because the probabilities must remain normalized over all bin–type pairs, so the objective is not separable in μ_{bm}. To avoid this, we apply the multinomial–Poisson

bioRxiv preprint doi: https://doi.org/10.64898/2026.04.18.719374; this version posted April 21, 2026. The copyright holder for this preprint (which was not certified by peer review) is the author/funder, who has granted bioRxiv a license to display the preprint in perpetuity. It is made available under aCC-BY 4.0 International license.

transformation [3]. Introduce an auxiliary scalar parameter $c_k \in \mathbb{R}$, define

$$\lambda_{bm}(\theta_k, c_k) := \exp(c_k)\mu_{bm}(\theta_k),$$

and consider the Poisson objective

$$\ell_{\text{Pois}}(\theta_k, c_k) = \sum_{b,m} \Big(\widetilde{Y}_{bm} \log \lambda_{bm} - \lambda_{bm}\Big). \tag{12}$$

Substituting $\lambda_{bm} = \exp(c_k)\mu_{bm}$ yields

$$\ell_{\text{Pois}}(\theta_k, c_k) = c_k \sum_{b,m} \widetilde{Y}_{bm} + \sum_{b,m} \widetilde{Y}_{bm} \log \mu_{bm} - \exp(c_k) \sum_{b,m} \mu_{bm}.$$

Maximizing over c_k gives

$$\exp(c_k^*) = \frac{\sum_{b,m} \widetilde{Y}_{bm}}{\sum_{b,m} \mu_{bm}}, \tag{13}$$

and substituting this back into the Poisson objective yields

$$\ell_{\text{Pois}}(\theta_k, c_k^*) = \sum_{b,m} \widetilde{Y}_{bm} \log \mu_{bm} - \Big(\sum_{b,m} \widetilde{Y}_{bm}\Big) \log\Big(\sum_{b,m} \mu_{bm}\Big) + C(\widetilde{Y}),$$

where $C(\widetilde{Y})$ is a constant that depends only on $\widetilde{Y}$. Comparing this expression with (11), we see that the profiled Poisson objective is equal to the multinomial objective up to an additive constant. Hence both objectives have the same maximizer in θ_k. Therefore, updating θ_k is equivalent to maximizing $\ell_{\text{Pois}}(\theta_k, c_k)$ with respect to both θ_k and c_k, and then retaining the optimal θ_k^*.

In MuTopia, the unnormalized mutation rate is

$$\mu_{bm}(\theta_k) = t_{bm} \exp\big(f_k(X_b) + g_{km}(X_b)\big) \tag{14}$$

so that

$$\log \lambda_{bm} = c_k + \log t_{bm} + f_k(X_b) + g_{km}(X_b).$$

Thus, the M-step for θ_k can be reformulated as maximizing a Poisson log-likelihood with offset $\log t_{bm}$, soft count response $\widetilde{Y}_{bm}$, and process-specific intercept c_k. The key advantage of this reformulation is that it replaces the normalized multinomial objective by a loss that is separable across bin–type pairs. In practice, this allows f_k and g_k to be fit using any model class that can be trained against a Poisson loss, including flexible estimators such as gradient boosted trees.

Updating the macro-scale effects f_k. Let θ_k^f denote the parameters determining the macro-scale effects of process k. As shown in the previous section, maximizing $\mathcal{L}$ with respect to θ_k^f is equivalent to maximizing the Poisson objective

$$\ell_{\text{Pois}}(\theta_k^f, c_k) = \sum_{b,m} \Big(\widetilde{Y}_{bm} \log \lambda_{bm} - \lambda_{bm}\Big) \tag{15}$$

with respect to both θ_k^f and c_k, and then retaining the optimal θ_k^f. Here,

$$\widetilde{Y}_{bm} = \sum_{n} Y_{nbm}\phi_{nbmk}$$

denotes the soft count assigned to process k in bin b and mutation type m, and

$$\log \lambda_{bm} = c_k + \log t_{bm} + f_k(X_b) + g_{km}(X_b),$$

where g_k is treated as fixed throughout this update.

bioRxiv preprint doi: https://doi.org/10.64898/2026.04.18.719374; this version posted April 21, 2026. The copyright holder for this preprint (which was not certified by peer review) is the author/funder, who has granted bioRxiv a license to display the preprint in perpetuity. It is made available under aCC-BY 4.0 International license.

To optimize this objective with respect to θ_k^f using standard machinery, it is convenient to rewrite it as a weighted Poisson loss whose rates depend on θ_k^f only through f_k. Since the macro-scale effects do not vary with mutation type, it is also natural to aggregate over mutation types. For fixed c_k, define

$$w_b = \exp(c_k) \sum_m t_{bm} \exp\big(g_{km}(X_b)\big), \qquad \widetilde{Y}_b = \frac{\sum_m \widetilde{Y}_{bm}}{w_b}. \tag{16}$$

Then

$$\ell_{\text{Pois}}(\theta_k^f, c_k) = \sum_b w_b \Big(\widetilde{Y}_b f_k(X_b) - \exp\big(f_k(X_b)\big)\Big) + \text{const}, \tag{17}$$

where const denotes terms independent of θ_k^f. Thus, for fixed c_k, updating f_k reduces to maximizing a weighted Poisson log-likelihood over bins, with sample weights w_b, responses $\widetilde{Y}_b$, and predictors X_b. This form of the loss allows us to fit f_k using the scikit-learn [4] implementation of histogram gradient-boosted tree (HGBT) regression [5] with a Poisson objective. In practice, when updating f_k, we restrict X_b to the macro-scale features X_b^{macro} (**Table 3**).

For fixed θ_k^f, the optimal c_k is given by the general update from (13). With the process topographies parameterized as in MuTopia, this reads

$$\exp(c_k) = \frac{\sum_{b,m} \widetilde{Y}_{bm}}{\sum_{b,m} t_{bm} \exp\big(f_k(X_b) + g_{km}(X_b)\big)}. \tag{18}$$

The updates for θ_k^f and c_k are alternated until convergence.

Incremental tree growth. Rather than fitting a new boosted tree model for f_k from scratch at every iteration, we instead maintain a single growing ensemble and append only a small number of additional trees at each update. This substantially improves training speed. We use this strategy both within the alternating updates of θ_k^f and c_k, and across successive iterations of the outer training loop over all variational and model parameters.

Specifically, let $f_k^{(0)}$ denote the current estimate of the macro-scale effects. To learn an additive update f_k, we optimize the Poisson objective (15) under the parameterization

$$\log \lambda_{bm} = c_k + \log t_{bm} + f_k^{(0)}(X_b) + f_k(X_b) + g_{km}(X_b).$$

Thus, the newly learned function f_k is interpreted as an increment to the current estimate $f_k^{(0)}$. As before, for fixed c_k and $f_k^{(0)}$, the objective can be rewritten as a weighted Poisson loss. The only difference is that the weights must now also absorb the contribution of the current macro-scale estimate. Define

$$w_b^{(0)} := \exp\big(f_k^{(0)}(X_b)\big)\, w_b, \qquad \widetilde{Y}_b^{(0)} := \frac{\sum_m \widetilde{Y}_{bm}}{w_b^{(0)}}.$$

Then

$$\ell_{\text{Pois}}(\theta_k^f, c_k) = \sum_b w_b^{(0)} \Big(\widetilde{Y}_b^{(0)} f_k(X_b) - \exp\big(f_k(X_b)\big)\Big) + \text{const}, \tag{19}$$

where const denotes terms independent of the additive update f_k. We fit this update using HGBT regression with a small number of boosting stages, and then update the macro-scale effects via

$$f_k^{(0)} \leftarrow f_k^{(0)} + f_k.$$

Repeated application of this procedure yields a single additive ensemble for the macro-scale effects, rather than a sequence of independently refit models.

bioRxiv preprint doi: https://doi.org/10.64898/2026.04.18.719374; this version posted April 21, 2026. The copyright holder for this preprint (which was not certified by peer review) is the author/funder, who has granted bioRxiv a license to display the preprint in perpetuity. It is made available under aCC-BY 4.0 International license.

Updating the spectra effects g_k. Let θ_k^g denote the parameters determining the spectra effects of process k. As shown above, maximizing $\mathcal{L}$ with respect to θ_k^g is equivalent to maximizing the Poisson objective

$$\ell_{\mathrm{Pois}}(\theta_k^g, c_k) = \sum_{b,m} \left(\widetilde{Y}_{bm} \log \lambda_{bm} - \lambda_{bm} \right) \tag{20}$$

with respect to both θ_k^g and c_k, and then retaining the optimal θ_k^g. Here,

$$\widetilde{Y}_{bm} = \sum_n Y_{nbm} \phi_{nbmk}$$

denotes the soft count assigned to process k in bin b and mutation type m, and

$$\log \lambda_{bm} = c_k + \log t_{bm} + f_k(X_b) + g_{km}(X_b),$$

where f_k is treated as fixed throughout this update.

Defining

$$w_{bm} = t_{bm} \exp\bigl(f_k(X_b)\bigr), \qquad \widetilde{Y}_{bm}^w = \frac{\widetilde{Y}_{bm}}{w_{bm}}, \tag{21}$$

we can rewrite the objective as

$$\ell_{\mathrm{Pois}}(\theta_k^g, c_k) = \sum_{b,m} w_{bm} \left(\widetilde{Y}_{bm}^w \bigl(c_k + g_{km}(X_b)\bigr) - \exp\bigl(c_k + g_{km}(X_b)\bigr) \right) + \mathrm{const}, \tag{22}$$

where const denotes terms independent of θ_k^g and c_k. Since $g_{km}(X_b)$ is linear in θ_k^g under the parameterization below, θ_k^g and c_k can be optimized jointly by weighted Poisson regression over bin–type pairs (b, m). Unlike for the non-linear macro-scale effects f_k, it is not necessary to alternate between their optimizations or absorb the auxiliary scalar parameter c_k into the weights and responses.

Structure of the spectra effects. We decompose the spectra effects for process k into baseline, meso-scale, and strand-orientation terms,

$$g_{km}(X_b) = g_{km}^{(0)} + g_{km}^{(1)}(X_b) + g_{km}^{(2)}(X_b). \tag{23}$$

Here, $g_k^{(0)}$ defines a baseline mutation spectrum for the process, $g_k^{(1)}$ captures meso-scale genomic modulation, and $g_k^{(2)}$ captures strand-dependent modulation.

To define these terms, let $X_b^{(1)} \in \mathbb{R}^{N_{\text{meso}}}$ denote the meso-scale features and let $X_b^{(2)} \in \{+, -, \varnothing\}^{N_{\text{strand}}}$ denote the strand-orientation features at bin b. For each mutation type $m \in \{1, \ldots, 192\}$, let

$$m' = m'(m) \in \{1, \ldots, 96\}$$

denote its C/T-centered representation, and let

$$s(m) \in \{+, -\}$$

denote its strand configuration, with $+$ for C/T-centered mutation types and $-$ for G/A-centered mutation types.

Baseline spectrum. The baseline spectrum is parameterized by a vector $a_k^{(0)} \in \mathbb{R}^{96}$, and is given by

$$g_{km}^{(0)} = a_{km'}^{(0)}. \tag{24}$$

Thus, the baseline spectrum is shared between each mutation type and its C/T-centered reverse complement.

bioRxiv preprint doi: https://doi.org/10.64898/2026.04.18.719374; this version posted April 21, 2026. The copyright holder for this preprint (which was not certified by peer review) is the author/funder, who has granted bioRxiv a license to display the preprint in perpetuity. It is made available under aCC-BY 4.0 International license.

Meso-scale effects. For the meso-scale effects, we introduce an unpenalized shared coefficient vector

$$a_k^{(1)} \in \mathbb{R}^{N_{\text{meso}}},$$

and an ℓ_1-penalized mutation-type-specific coefficient matrix

$$A_k^{(1)} \in \mathbb{R}^{N_{\text{meso}} \times 96}.$$

The resulting contribution is

$$g_{km}^{(1)}(X_b) = {X_b^{(1)}}^\top a_k^{(1)} + \left((X_b^{(1)})^\top A_k^{(1)}\right)_{m'}. \tag{25}$$

Thus, each meso-scale feature contributes both a mutation-type-independent shift and a mutation-type-specific deviation indexed by the C/T-centered mutation type.

Strand effects. For the strand effects, we introduce unpenalized shared coefficient vectors

$$a_k^{(2)},\ \bar{a}_k^{(2)} \in \mathbb{R}^{N_{\text{strand}}},$$

together with ℓ_1-penalized mutation-type-specific coefficient matrices

$$A_k^{(2)},\ \bar{A}_k^{(2)} \in \mathbb{R}^{N_{\text{strand}} \times 192}.$$

For each strand feature i, the contribution depends on whether the strand annotation at bin b matches the strand configuration of mutation type m. Writing $x_{bi}^{(2)} \in \{+, -, \varnothing\}$ for the i-th strand feature, we define

$$g_{km}^{(2)}(X_b) = \sum_{i:\, x_{bi}^{(2)} \neq \varnothing} \begin{cases} a_{ki}^{(2)} + A_{kim}^{(2)}, & \text{if } x_{bi}^{(2)} = s(m), \\ \bar{a}_{ki}^{(2)} + \bar{A}_{kim}^{(2)}, & \text{if } x_{bi}^{(2)} \neq s(m). \end{cases} \tag{26}$$

Unlike the meso-scale interaction coefficients, the strand-specific interaction terms $A_{kim}^{(2)}$ and $\bar{A}_{kim}^{(2)}$ are indexed by the full mutation type $m \in \{1, \ldots, 192\}$, since strand orientation distinguishes reverse-complement mutation types.

Table 1 illustrates the coefficients chosen by a summand of (26) for a representative mutation type and its reverse complement: the two receive different coefficients depending on their orientation relative to the strand feature.

mutation type m	$s(m)$	strand feature $x_{bi}^{(2)}$		
		$+$	$-$	$\varnothing$
$G[C>G]T$	$+$	$a_{ki}^{(2)} + A_{kim}^{(2)}$	$\bar{a}_{ki}^{(2)} + \bar{A}_{kim}^{(2)}$	0
$A[G>C]C$	$-$	$\bar{a}_{ki}^{(2)} + \bar{A}_{kim}^{(2)}$	$a_{ki}^{(2)} + A_{kim}^{(2)}$	0

Table 1: Strand-dependent coefficients. Coefficients selected for a mutation type and its reverse-complement. The two receive different coefficients depending on whether the strand feature matches the mutation orientation.

Optimization of the spectra effects g_k. Under the parameterization above, $\log \lambda_{bm}$ is linear in the parameters

$$\theta_k^g = \left(a_k^{(0)}, a_k^{(1)}, A_k^{(1)}, a_k^{(2)}, A_k^{(2)}, \bar{a}_k^{(2)}, \bar{A}_k^{(2)}\right)$$

and c_k. Therefore, for fixed f_k, updating g_k is equivalent to solving a weighted Poisson generalized linear model with responses $\widetilde{Y}_{bm}^{w}$, weights w_{bm}, and linear predictor $c_k + g_{km}(X_b)$. We solve this problem using regularized itera-

bioRxiv preprint doi: https://doi.org/10.64898/2026.04.18.719374; this version posted April 21, 2026. The copyright holder for this preprint (which was not certified by peer review) is the author/funder, who has granted bioRxiv a license to display the preprint in perpetuity. It is made available under aCC-BY 4.0 International license.

tively reweighted least squares in the style of `glmnet` [6], applying ℓ_1-penalization only to the mutation-type-specific interaction terms $A_k^{(1)}$, $A_k^{(2)}$, and $\bar{A}_k^{(2)}$, while leaving the shared coefficients and baseline spectrum unpenalized.

In summary, the spectra effects model combines an unpenalized baseline spectrum $a_k^{(0)}$, unpenalized shared feature effects $\left(a_k^{(1)}, a_k^{(2)}, \bar{a}_k^{(2)}\right)$, and sparse mutation-type-specific interaction terms $\left(A_k^{(1)}, A_k^{(2)}, \bar{A}_k^{(2)}\right)$. This design allows the model to capture broad systematic shifts in mutational spectrum while only introducing mutation-type-specific deviations from the baseline spectrum when strongly supported by the data.

Scalable parameter estimation via stochastic variational inference

Algorithm 1 summarizes the overall coordinate-ascent structure of MuTopia model fitting, while Algorithm 2 makes the corresponding full-batch variational E-step and M-step updates explicit. In the full-batch procedure, all variational and model parameters are updated using the complete dataset at every iteration. Although these updates monotonically increase the ELBO, this becomes computationally expensive for large datasets.

To improve scalability, we therefore use stochastic variational inference (SVI; Algorithm 3), following the general framework of Hoffman et al. [7]. At each iteration, we subsample a set of genomic bins and restrict the mutation counts and features to those bins. We then apply the same variational E-step and M-step structure as in the full-batch algorithm, but on the subsampled data. To obtain an unbiased estimate of the corresponding full-data update, the minibatch contribution to the variational parameter β_n is rescaled by the inverse subsampling rate. The resulting stochastic parameter estimates are then merged with the current global parameters using the learning rate $\rho_i = (i+1)^{-0.5}$. Under standard conditions for stochastic approximation, these updates converge to a local optimum of the variational objective provided that they yield unbiased estimates of the natural gradient [7]. In our setting, this condition is satisfied by bin subsampling together with the rescaling step above. After convergence, sample-specific variational parameters can be obtained by a final E-step on the full data using the fitted global parameters. In practice, SVI substantially reduces training time and often yields better solutions than the full-batch procedure.

Algorithm 1 MuTopia — coordinate ascent overview.

Input: mutation counts $Y \in \mathbb{N}^{N_{\text{samples}} \times N_{\text{bins}} \times N_{\text{types}}}$, genomic features $X \in \mathbb{R}^{N_{\text{bins}} \times N_{\text{features}}}$
Init: model parameters θ, α; variational parameters ϕ, β
while not converged **do**
  $\phi, \beta \leftarrow$ E-step$(Y, X;\, \theta, \alpha)$ ▷ Eq. 7, 8
  $\theta, \alpha \leftarrow$ M-step$(Y, X;\, \phi, \beta)$ ▷ Eq. 9, 17, 22
end while
return $\theta, \alpha, \beta, \phi$

bioRxiv preprint doi: https://doi.org/10.64898/2026.04.18.719374; this version posted April 21, 2026. The copyright holder for this preprint (which was not certified by peer review) is the author/funder, who has granted bioRxiv a license to display the preprint in perpetuity. It is made available under aCC-BY 4.0 International license.

Algorithm 2 MuTopia — full-batch coordinate ascent variational inference.

Input: mutation counts $Y \in \mathbb{N}^{N_{\text{samples}} \times N_{\text{bins}} \times N_{\text{types}}}$, genomic features $X \in \mathbb{R}^{N_{\text{bins}} \times N_{\text{features}}}$
Initialize: model parameters $\alpha, \theta = \{\theta_k^f, \theta_k^g\}_{k=1}^K$
Initialize: variational parameters β_n for all n
while not converged **do**
  Variational E-step: update variational parameters
  for sample $n = 1, \ldots, N_{\text{samples}}$ **do**
    initialize β_n (e.g. from previous iteration)
    repeat
      for process $k = 1, \ldots, K$ **do**
        $\widetilde{\pi}_{nk} = \exp\big(\psi(\beta_{nk}) - \psi(\sum_{k'} \beta_{nk'})\big)$ ▷ Eq. 3
      end for
      for all observed bin–type pairs (b, m) in sample n **do**
        $\phi_{nbmk} \propto p(b, m \mid z = k, \theta)\, \widetilde{\pi}_{nk}$ ▷ Eq. 7
      end for
      for process $k = 1, \ldots, K$ **do**
        $\beta_{nk} \leftarrow \alpha_k + \sum_{b,m} Y_{nbm}\phi_{nbmk}$ ▷ Eq. 8
      end for
    until β_n and ϕ_n converge
  end for
  Variational M-step: update global model parameters
  update α by Newton–Raphson ▷ Eq. 9
  for process $k = 1, \ldots, K$ **do**
    compute soft counts $\widetilde{Y}_{bm}^{(k)} \leftarrow \sum_n Y_{nbm}\phi_{nbmk}$ ▷ Eq. 10
    Update macro-scale effects f_k
    initialize c_k from previous iteration
    repeat
      form weighted Poisson problem over bins ▷ Eq. 17
      update the HGBT model parameters θ_k^f
      update c_k ▷ Eq. 18
    until θ_k^f and c_k converge
    Update spectra effects g_k
    form weighted Poisson regression problem over bin–type pairs ▷ Eq. 22
    update θ_k^g and c_k jointly by regularized IRLS
  end for
end while
return $\theta, \alpha, \beta, \phi$

Algorithm 3 MuTopia — stochastic variational inference.

Input: mutation counts $Y \in \mathbb{N}^{N_{\text{samples}} \times N_{\text{bins}} \times N_{\text{types}}}$, genomic features $X \in \mathbb{R}^{N_{\text{bins}} \times N_{\text{features}}}$, subsample rate $s \in (0, 1)$
Init: model parameters θ, α; iteration $i \leftarrow 1$
while not converged **do**
  Sample a subset of bins $\mathcal{B} \subseteq \{1, \ldots, N_{\text{bins}}\}$ with inclusion probability s
  $\dot{Y} \leftarrow Y_{:\mathcal{B},:}, \quad \dot{X} \leftarrow X_{\mathcal{B},:}$
  $\dot{\phi}, \dot{\beta} \leftarrow$ Variational E-step$(\dot{Y}, \dot{X};\, \theta, \alpha)$ ▷ Alg. 2
  $\dot{\beta}_n \leftarrow \alpha + \frac{1}{s}\big(\dot{\beta}_n - \alpha\big) \quad \forall n$
  $\dot{\theta}, \dot{\alpha} \leftarrow$ Variational M-step$(\dot{Y}, \dot{X};\, \dot{\phi}, \dot{\beta})$ ▷ Alg. 2
  $\rho \leftarrow (i + 1)^{-0.5}$
  $\theta \leftarrow (1 - \rho)\, \theta + \rho\, \dot{\theta}$
  $\alpha \leftarrow (1 - \rho)\, \alpha + \rho\, \dot{\alpha}$
  $i \leftarrow i + 1$
end while
return θ, α

bioRxiv preprint doi: https://doi.org/10.64898/2026.04.18.719374; this version posted April 21, 2026. The copyright holder for this preprint (which was not certified by peer review) is the author/funder, who has granted bioRxiv a license to display the preprint in perpetuity. It is made available under aCC-BY 4.0 International license.

Feature selection from ENCODE

We implemented a fully automated strategy to build tissue-matched corpora of genetic and epigenetic features for each dataset used in our study, following the AlphaGenome [8] approach of prioritizing samples based on their ENCODE-assessed quality control metrics. Briefly, we fetched unperturbed ENCODE experiments for DNase-seq, ATAC-seq, Whole-genome Bisulfite Sequencing (WGBS), Histone ChIP-seq, Repli-seq, Poly-A plus RNA-seq, and total RNA-seq assays. Using the ENCODE audit metadata, we filtered out experiments that failed to meet QC, read length, or fraction of reads in peaks (FRiP) thresholds, as outlined in AlphaGenome. We then assigned each experiment a quality score between -1 (FAIL) and 4 (PASS). Next, we grouped the experiments by biosample ontology term and assay. For each group, we chose the single most concordant experiment that best satisfied the following criteria:

1. Oldest life stage ("adult" prioritized over "child")
2. The description contained none of the terms "genetically modified", "arrested", or "treated".
3. Maximized quality score.
4. Used paired-end reads.
5. Conducted in tissue or primary cells.
6. Did not measure a subcellular fraction.

For experiments that met those criteria, we then selected the one with the highest FRiP, if applicable, or the most recently conducted.

Finally, for each tumor type in the PCAWG cohort, we specified a ranked list of Uberon tissue ontology terms [9] (**Table 2**). To choose the best-matched experiment to represent a tumor cell type, we used the following prioritization heuristic, selecting the experiment that maximally satisfied the conditions:

1. Life stage was "adult".
2. Quality score exceeded 3.
3. Highest rank of tissue ontology term.
4. Highest quality score.

bioRxiv preprint doi: https://doi.org/10.64898/2026.04.18.719374; this version posted April 21, 2026. The copyright holder for this preprint (which was not certified by peer review) is the author/funder, who has granted bioRxiv a license to display the preprint in perpetuity. It is made available under aCC-BY 4.0 International license.

TumorType	Tissue terms (ranked)	Repli-seq terms
Bladder-TCC	UBERON:0001255; UBERON:0001259; UBERON:0005033; CL:2000040	EFO:0001187
Cervix-All	UBERON:0000995; EFO:0002791	EFO:0002791
ColoRect-AdenoCA	UBERON:0000317; UBERON:0004992; UBERON:0001159; UBERON:0001157; UBERON:0008971	EFO:0001187
Eso-AdenoCA	UBERON:0001043; UBERON:0002469; UBERON:0004648	EFO:0001187
Head-SCC	UBERON:0006920	EFO:0001196
Breast-All	UBERON:0008367; UBERON:0000310; CL:0002327	EFO:0001203
Kidney-All	UBERON:0002113; UBERON:0004538; UBERON:0004539; UBERON:0001225; CL:0002518; CL:1000510; CL:1000892; UBERON:0001255	EFO:0001187
Liver-HCC	UBERON:0002107; UBERON:0001115; UBERON:0001114	EFO:0001187
Lung-All	UBERON:0002048; UBERON:0002168; UBERON:0002167; UBERON:0008953; UBERON:0008952; UBERON:0002170; UBERON:0002171	EFO:0001196
Ovary-AdenoCA	UBERON:0000992	EFO:0001203
Panc-AdenoCA	UBERON:0001264; UBERON:0001150	EFO:0001187
Prost-AdenoCA	UBERON:0002367; CL:0002231	EFO:0001203
Skin-Melanoma	CL:1000458; UBERON:0002097; UBERON:0001003; UBERON:0004264; UBERON:0036149	EFO:0001196
Stomach-AdenoCA	UBERON:0000945	EFO:0001187
Uterus-AdenoCA	UBERON:0000995; EFO:0002791	EFO:0002791

Table 2: Uberon cell type ontology terms — used to query ENCODE experiments for each tumor type.

For replication timing data, we utilized the best-matched cell line in which each phase had been assayed. We used the *LiftOver* [10] tool to convert the hg19-aligned Repli-seq "percentage normalized signal" files to hg38 coordinates in line with the rest of our analysis.

All together, **Table 3** summarizes how these genomic features were used in the MuTopia model. Macro features (continuous-valued) capture megabase-scale variation in mutation rate. Meso and strand features modulate the mutation type distribution within a bin.

Software and implementation

MuTopia was written in Python, with numerical operations implemented using *numpy, scipy, sparse* and *numba* [12–14]. Hyperparameter optimization was managed using *optuna* [15]. Data organization was facilitated using *xArray*, *netCDF4*, and *Pandas* [16–18]. Dataset configuration and construction were implemented using *pydantic*, *pyyaml*, and *luigi*. Plotting and analyses were conducted with *matplotlib*, *jupyter*, and *pygenometracks* [19, 20].

bioRxiv preprint doi: https://doi.org/10.64898/2026.04.18.719374; this version posted April 21, 2026. The copyright holder for this preprint (which was not certified by peer review) is the author/funder, who has granted bioRxiv a license to display the preprint in perpetuity. It is made available under aCC-BY 4.0 International license.

Feature	Biological signal	Feature type	Tissue-matched	Assay/Source	Normalization	Affects binning	Domain
Gene expression	Transcriptional activity	Macro	Yes	RNA-seq	Log1p-CPTM	No	continuous
Chromatin activity	Diffuse chromatin accessibility	Macro	Yes	DNase-seq	Log1p-CPTM	No	continuous
Repli-seq (G1b, S1–S4, G2)	Replication timing phases	Macro	Yes	Repli-seq	quantile	No	continuous
H3K27ac, H3K4me1, H3K4me3	Active enhancers and promoters	Macro	Yes	ChIP-seq	Log1p-CPTM	No	continuous
H3K36me3, H3K27me3, H3K9me3	Heterochromatin marks	Macro	Yes	ChIP-seq	Log1p-CPTM	No	continuous
CpG methylation fraction	DNA methylation	Macro	Yes	WGBS	standardized	No	continuous
GC content	Nucleotide composition	Macro	No	Reference genome	standardized	No	continuous
Repeat element fraction	Repeat element density	Macro	No	Reference genome	standardized	No	continuous
ATAC accessibility	Focal open chromatin regions	Meso	Yes	ATAC-seq	quantile	Yes	binary
Gene strand	Transcription orientation	Strand	No	MANE annotation [11]	—	Yes	$\{+, -, \varnothing\}$
Replication strand	Replication fork direction	Strand	No†	Repli-seq (MCF7)	—	Yes	$\{+, -, \varnothing\}$

Table 3: Genomic feature overview. Features used in the MuTopia model, grouped by feature type (macro, meso, strand). Log1p-CPTM: log1p-counts per ten million; quantile: quantile normalization; Repeat element fraction is the fraction of soft-masked bases in the reference genome for a given bin. †Replication strand was derived from MCF7 Repli-seq and is not tissue-matched for non-breast tumor types.

bioRxiv preprint doi: https://doi.org/10.64898/2026.04.18.719374; this version posted April 21, 2026. The copyright holder for this preprint (which was not certified by peer review) is the author/funder, who has granted bioRxiv a license to display the preprint in perpetuity. It is made available under aCC-BY 4.0 International license.

References

1. Blei, D. M., Kucukelbir, A. & McAuliffe, J. D. Variational inference: A review for statisticians. *Journal of the American statistical Association* **112,** 859–877 (2017).
2. Blei, D., Ng, A. & Jordan, M. *Latent Dirichlet Allocation* in *Advances in Neural Information Processing Systems* **14** (MIT Press, 2001).
3. Baker, S. G. The Multinomial-Poisson Transformation. *Journal of the Royal Statistical Society. Series D (The Statistician)* **43,** 495–504 (1994).
4. Pedregosa, F. *et al. Scikit-learn: Machine Learning in Python* 2018. arXiv: `1201.0490[cs]`.
5. Ke, G. *et al. LightGBM: A Highly Efficient Gradient Boosting Decision Tree* in *Advances in Neural Information Processing Systems* **30** (Curran Associates, Inc., 2017).
6. Friedman, J., Hastie, T. & Tibshirani, R. Regularization Paths for Generalized Linear Models via Coordinate Descent. *Journal of Statistical Software* **33** (2010).
7. Hoffman, M. D., Blei, D. M., Wang, C. & Paisley, J. Stochastic Variational Inference. *Journal of Machine Learning Research* **14,** 1303–1347 (2013).
8. Avsec, Ž. *et al. AlphaGenome: advancing regulatory variant effect prediction with a unified DNA sequence model* Pages: 2025.06.25.661532 Section: New Results. 2025.
9. Mungall, C. J., Torniai, C., Gkoutos, G. V., Lewis, S. E. & Haendel, M. A. Uberon, an integrative multi-species anatomy ontology. *Genome Biology* **13,** R5 (2012).
10. Hinrichs, A. S. *et al.* The UCSC Genome Browser Database: update 2006. *Nucleic Acids Research* **34,** D590–D598 (suppl_1 2006).
11. Morales, J. *et al.* A joint NCBI and EMBL-EBI transcript set for clinical genomics and research. *Nature* **604,** 310–315 (2022).
12. Harris, C. R. *et al.* Array programming with NumPy. *Nature* **585,** 357–362 (2020).
13. Virtanen, P. *et al.* SciPy 1.0: fundamental algorithms for scientific computing in Python. *Nature Methods* **17,** 261–272 (2020).
14. Lam, S. K., Pitrou, A. & Seibert, S. *Numba: a LLVM-based Python JIT compiler* in *Proceedings of the Second Workshop on the LLVM Compiler Infrastructure in HPC* (Association for Computing Machinery, New York, NY, USA, 2015), 1–6.
15. Akiba, T., Sano, S., Yanase, T., Ohta, T. & Koyama, M. *Optuna: A Next-generation Hyperparameter Optimization Framework* arXiv.org. `https://arxiv.org/abs/1907.10902v1` (2025).
16. Hoyer, S. & Hamman, J. xarray: N-D labeled Arrays and Datasets in Python | Journal of Open Research Software (2017).
17. Rew, R. & Davis, G. NetCDF: an interface for scientific data access. *IEEE Computer Graphics and Applications* **10,** 76–82 (1990).
18. Pandas development team, T. *pandas-dev/pandas: Pandas* version latest. 2020.
19. Hunter, J. D. Matplotlib: A 2D graphics environment. *Computing in Science & Engineering* **9,** 90–95 (2007).
20. Lopez-Delisle, L. *et al.* pyGenomeTracks: reproducible plots for multivariate genomic datasets. *Bioinformatics* **37,** 422–423 (2021).

bioRxiv preprint doi: https://doi.org/10.64898/2026.04.18.719374; this version posted April 21, 2026. The copyright holder for this preprint (which was not certified by peer review) is the author/funder, who has granted bioRxiv a license to display the preprint in perpetuity. It is made available under aCC-BY 4.0 International license.

Supplementary figures

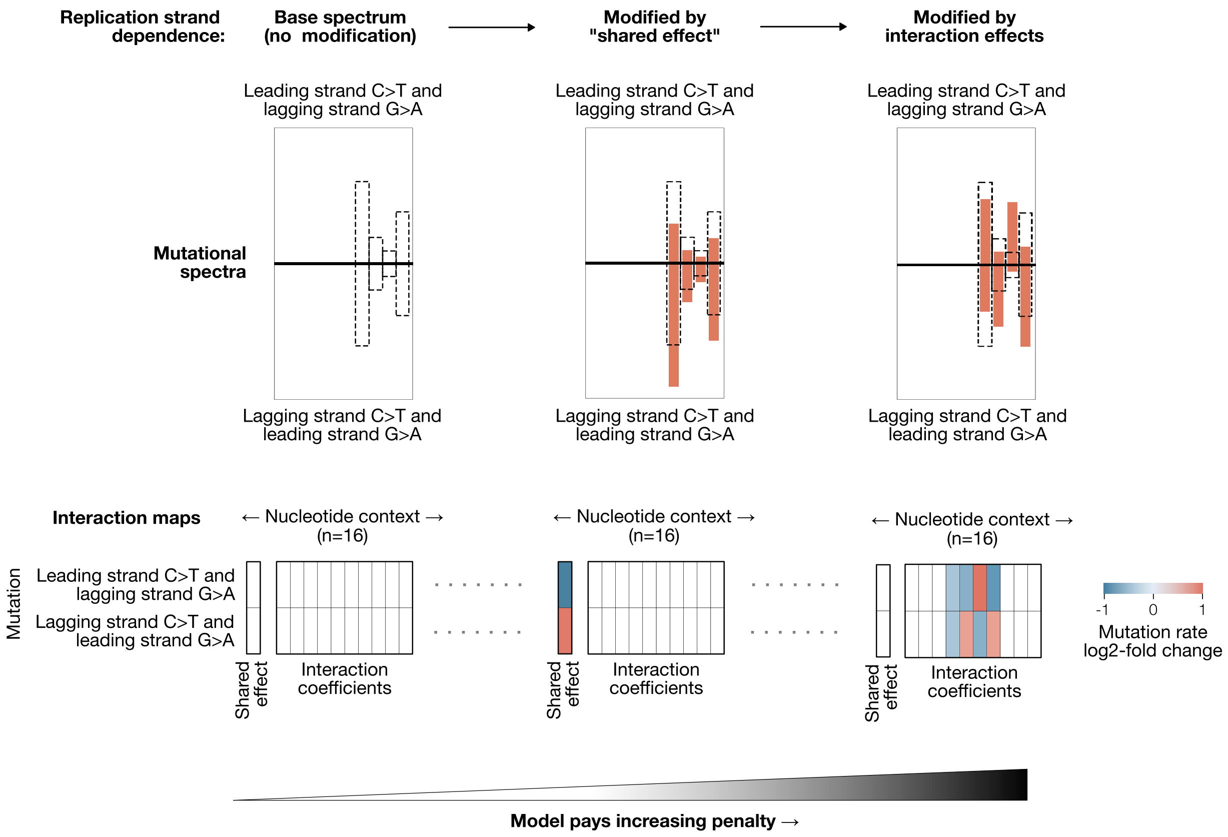

Supplementary Figure 1: Strand effect parameterization and regularization strategy. MuTopia learns context-dependent signatures in which mutation rates are modified by local genomic state on a per-mutation-type basis. To reduce the variance of the model when fitting these complex signatures, we introduce a hierarchical method for learning sparse “interaction maps”. First, no context dependence incurs no regularizing penalty (left). Then, uniform scaling across all mutation types (middle) is favored over the fitting of type-specific interactions (right).

bioRxiv preprint doi: https://doi.org/10.64898/2026.04.18.719374; this version posted April 21, 2026. The copyright holder for this preprint (which was not certified by peer review) is the author/funder, who has granted bioRxiv a license to display the preprint in perpetuity. It is made available under aCC-BY 4.0 International license.

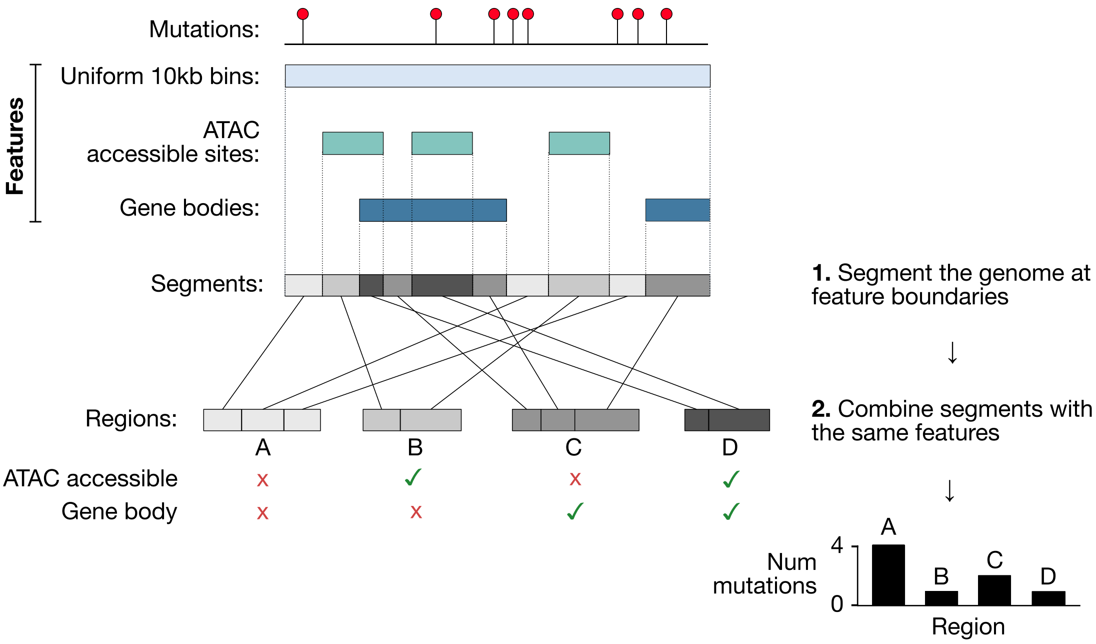

Supplementary Figure 2: MuTopia genome-binning and mutation aggregation strategy. For tractable parameter updates, we locally aggregate mutation counts into discontinuous bins that share the same locus features. The resolution of the model (in this case 10kb) determines the maximum distance at which any two mutations may be aggregated. The macro-scale bins are segmented according to the intersection of any underlying discrete genomic features. Then, mutations within segments which intersect the same sets of features are pooled. The segment groups are indicated by color.

bioRxiv preprint doi: https://doi.org/10.64898/2026.04.18.719374; this version posted April 21, 2026. The copyright holder for this preprint (which was not certified by peer review) is the author/funder, who has granted bioRxiv a license to display the preprint in perpetuity. It is made available under aCC-BY 4.0 International license.

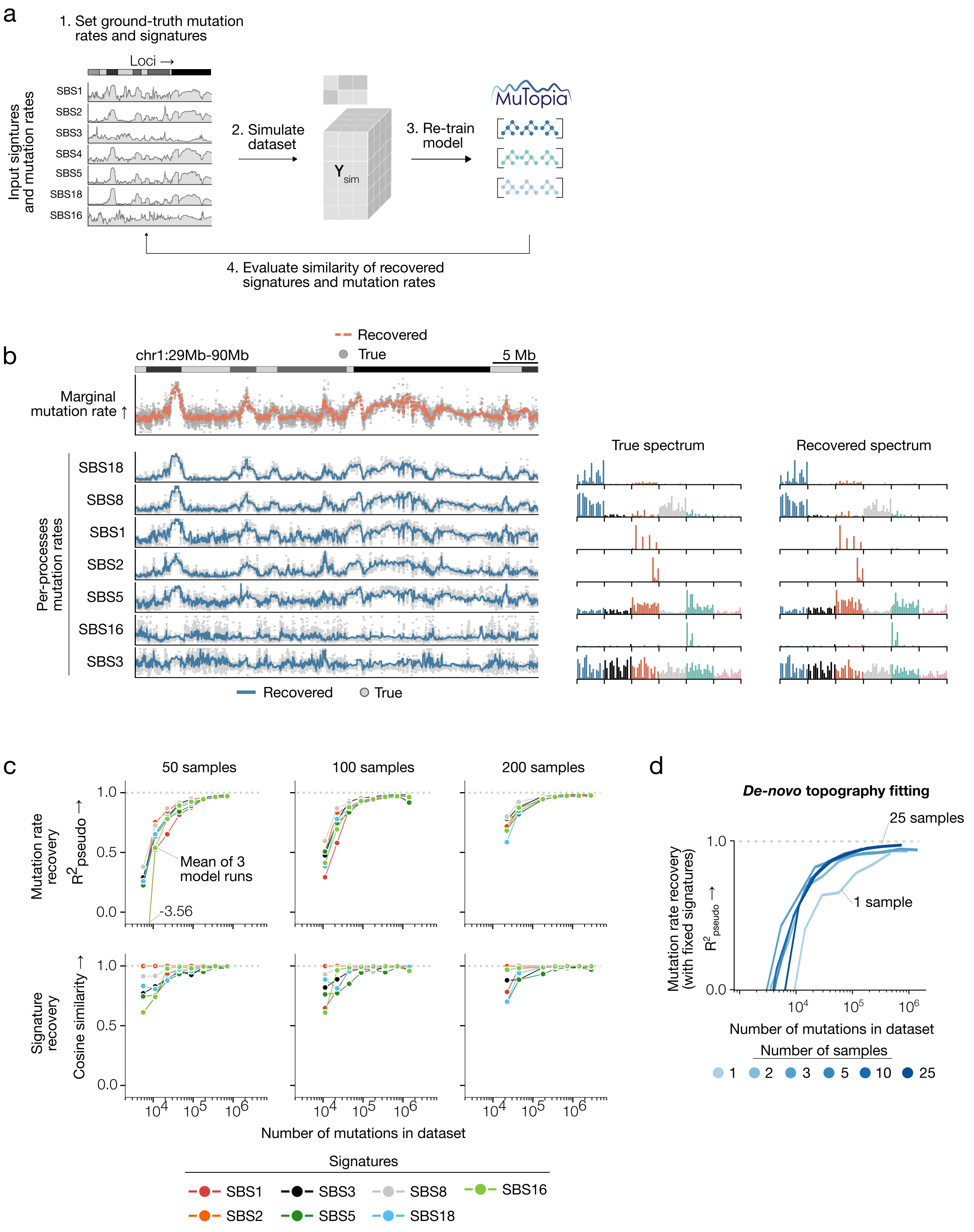

Supplementary Figure 3: Simulation-based benchmarking results. a) Schematic of the simulation-based evaluation strategy. Using realistic components fit from real breast cancer data, we sampled new datasets while varying the process contributions and burdens. We trained new models on these simulated datasets, then compared the similarity between the extracted components and those input into the simulation using pseudo-R^2 between mutation rate distributions and cosine similarity between spectra. **b)** The recovered mutational spectra and mutation rate distributions were compared to input data in unseen regions of the genome. **c)** Mutation rate and spectrum recovery quality, broken down by dataset size and by input signature for varying numbers of simulated samples. **d)** Performance on *de novo* topography fitting while fixing signatures.

bioRxiv preprint doi: https://doi.org/10.64898/2026.04.18.719374; this version posted April 21, 2026. The copyright holder for this preprint (which was not certified by peer review) is the author/funder, who has granted bioRxiv a license to display the preprint in perpetuity. It is made available under aCC-BY 4.0 International license.

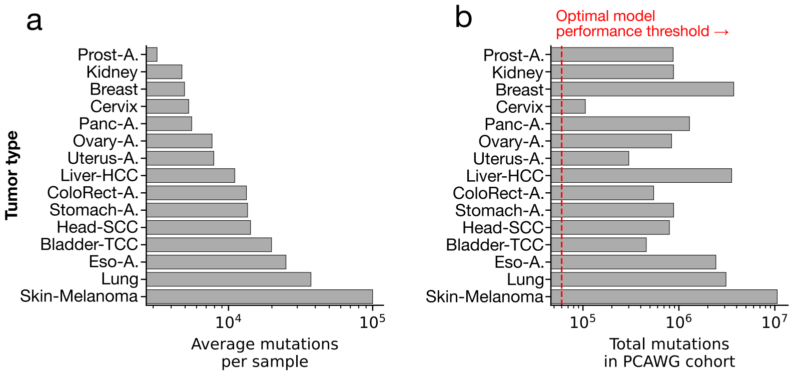

Supplementary Figure 4: Pan-cancer dataset properties. a) Average number of mutations per sample in the dataset. **b)** Total number of mutations per tumor type.

bioRxiv preprint doi: https://doi.org/10.64898/2026.04.18.719374; this version posted April 21, 2026. The copyright holder for this preprint (which was not certified by peer review) is the author/funder, who has granted bioRxiv a license to display the preprint in perpetuity. It is made available under aCC-BY 4.0 International license.

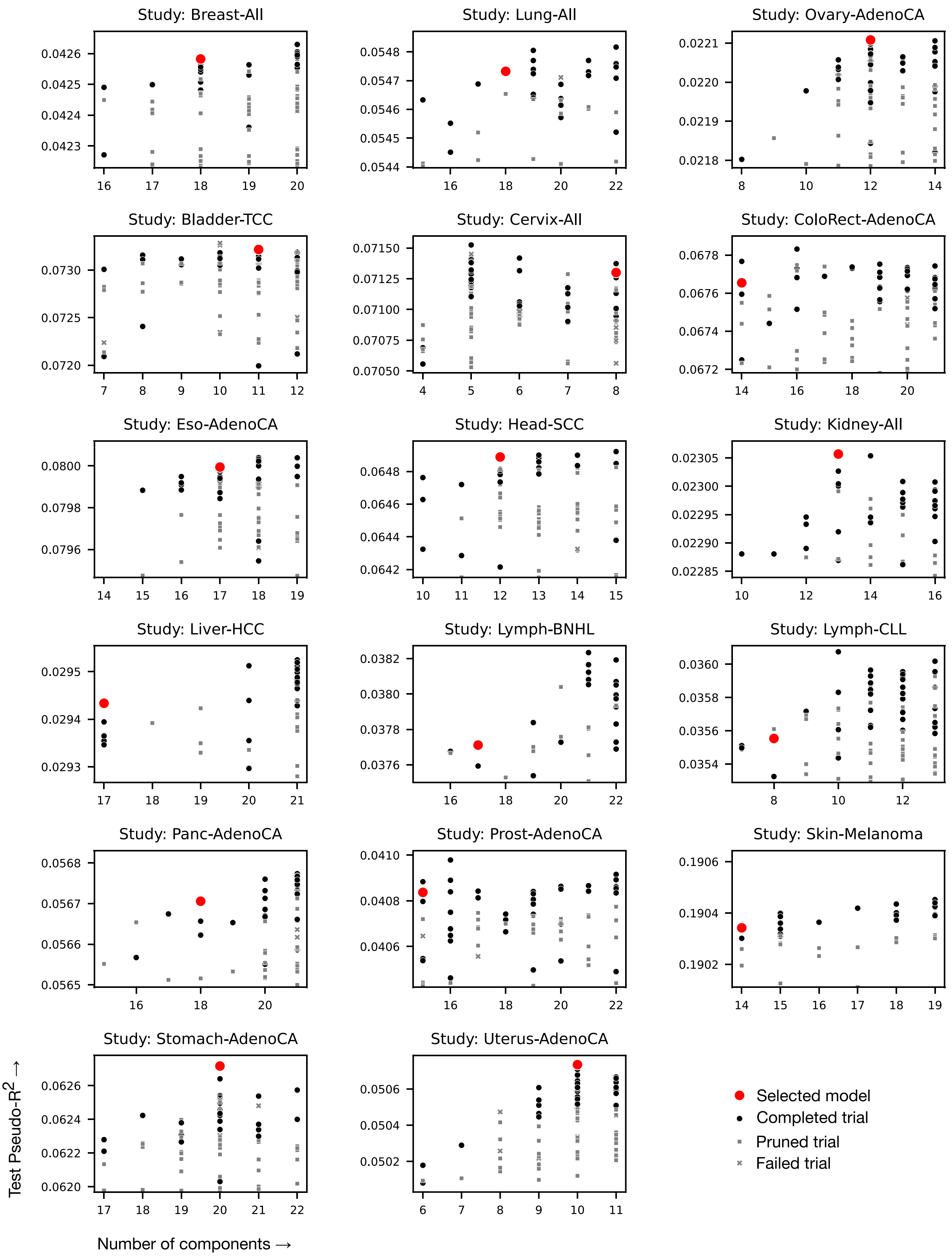

Supplementary Figure 5: Pan-cancer hyperparameter optimization results. Number of components versus score on held-out chromosome 2 for the hyperparameter optimization "studies" performed on each tumor type.

bioRxiv preprint doi: https://doi.org/10.64898/2026.04.18.719374; this version posted April 21, 2026. The copyright holder for this preprint (which was not certified by peer review) is the author/funder, who has granted bioRxiv a license to display the preprint in perpetuity. It is made available under aCC-BY 4.0 International license.

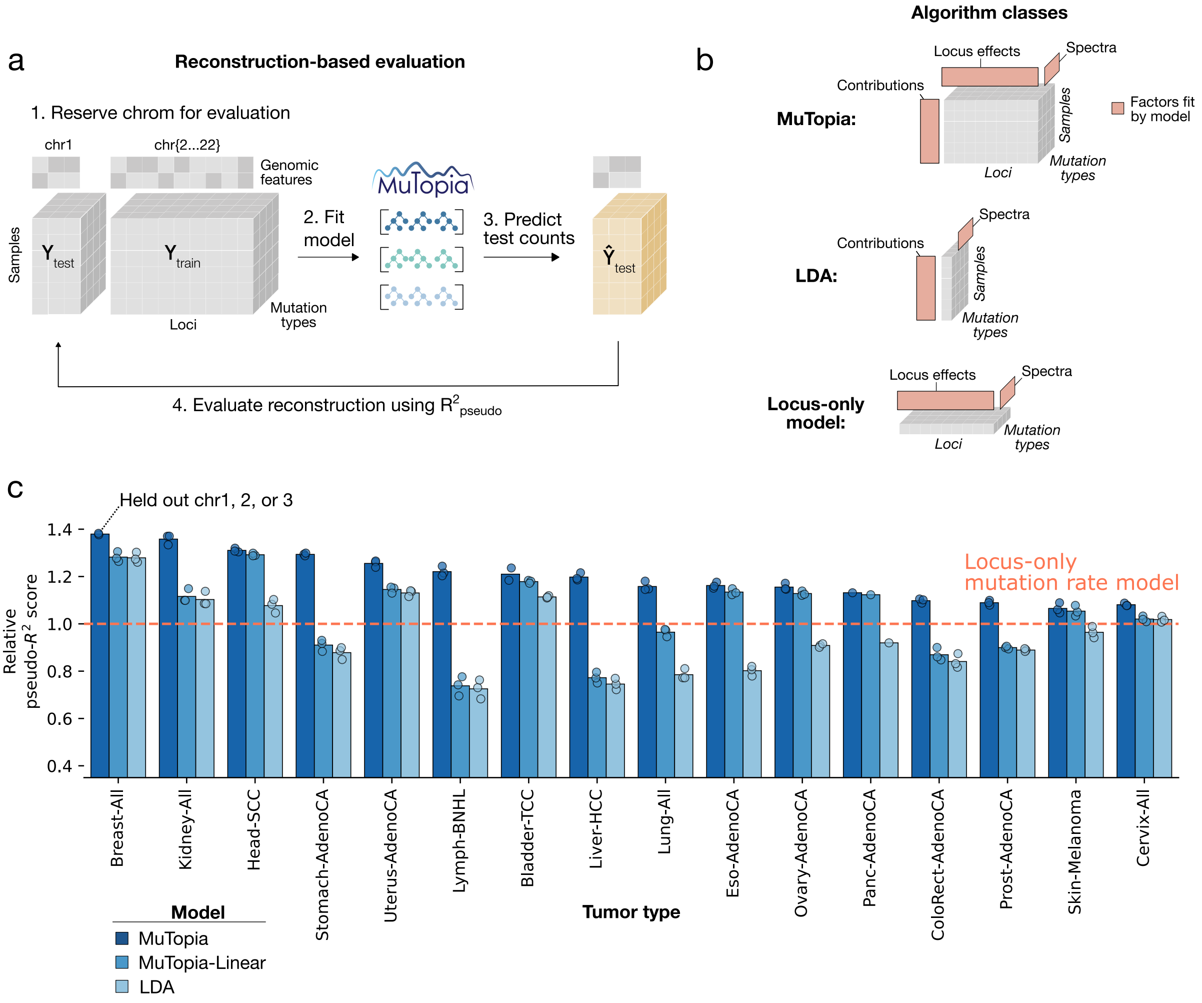

Supplementary Figure 6: Reconstruction-based evaluation and model ablation results. a) Reconstruction-based evaluation method. Test data were reserved by chromosome, then imputed from a trained model. **b)** Algorithm classes evaluated in benchmarking tests. MuTopia fits three factors: process contributions, locus effects, and spectra. LDA/NMF omits locus effects, while "locus only" omits sample-level contributions. **c)** Results from reconstruction-based evaluation using the relative pseudo-R^2 metric. Bars show the full and ablated versions of the MuTopia model. The dashed line shows the likelihood of the data using a baseline locus-based mutation rate model.

bioRxiv preprint doi: https://doi.org/10.64898/2026.04.18.719374; this version posted April 21, 2026. The copyright holder for this preprint (which was not certified by peer review) is the author/funder, who has granted bioRxiv a license to display the preprint in perpetuity. It is made available under aCC-BY 4.0 International license.

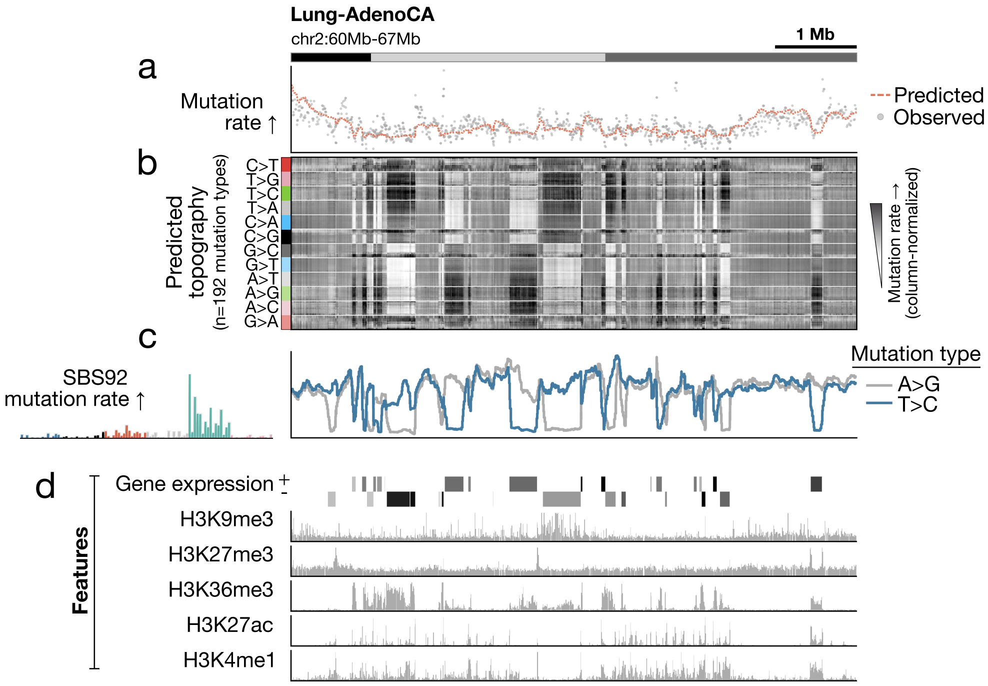

Supplementary Figure 7: Mutational topography at the intermediate scale. **a)** Observed versus predicted mutation rates on a held-out section of the genome. **b)** Mutational topography prediction. **c)** Predicted mutation rate profiles of A>G versus T>C mutations originating from SBS92. **d)** Representative genomic features driving variation in mutation rates at this scale.

bioRxiv preprint doi: https://doi.org/10.64898/2026.04.18.719374; this version posted April 21, 2026. The copyright holder for this preprint (which was not certified by peer review) is the author/funder, who has granted bioRxiv a license to display the preprint in perpetuity. It is made available under aCC-BY 4.0 International license.

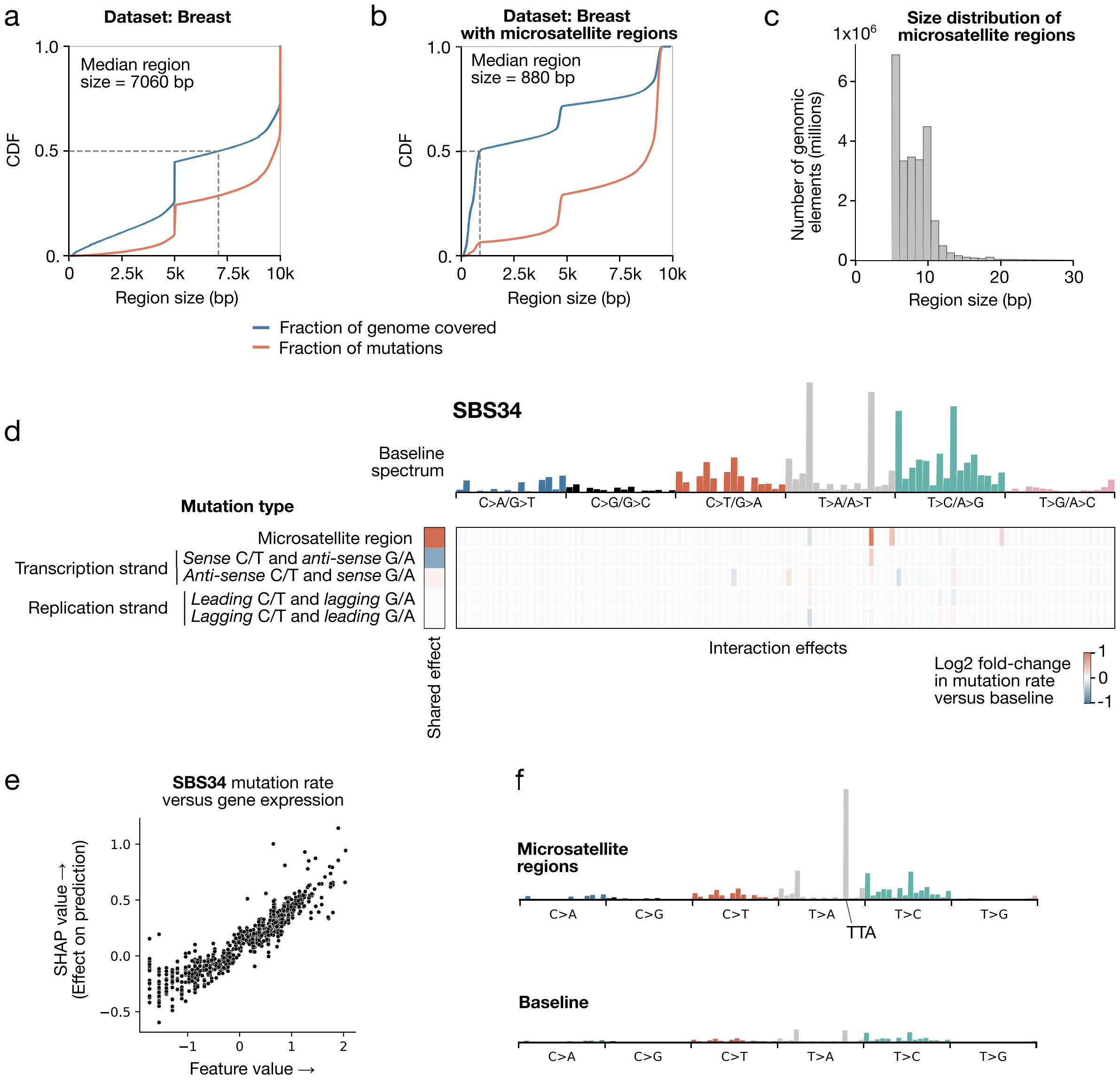

Supplementary Figure 8: *Meso*-scale analysis of mutagenesis at microsatellite sites. a) For the breast dataset, cumulative distribution functions of fraction of genome covered by segmented regions versus the fraction of mutations those bins contain, sorted by region size. **b)** Same as (a), except with the addition of *meso*-scale features indicating microsatellite regions. **c)** Size distribution of microsatellite regions. **d)** Context interaction map for signature SBS34. **e)** Gene expression Shapley value versus feature value for signature SBS34. **f)** SBS34 signature mutational spectra in a “baseline” genomic context (bottom), and in microsatellite regions (top).

bioRxiv preprint doi: https://doi.org/10.64898/2026.04.18.719374; this version posted April 21, 2026. The copyright holder for this preprint (which was not certified by peer review) is the author/funder, who has granted bioRxiv a license to display the preprint in perpetuity. It is made available under aCC-BY 4.0 International license.

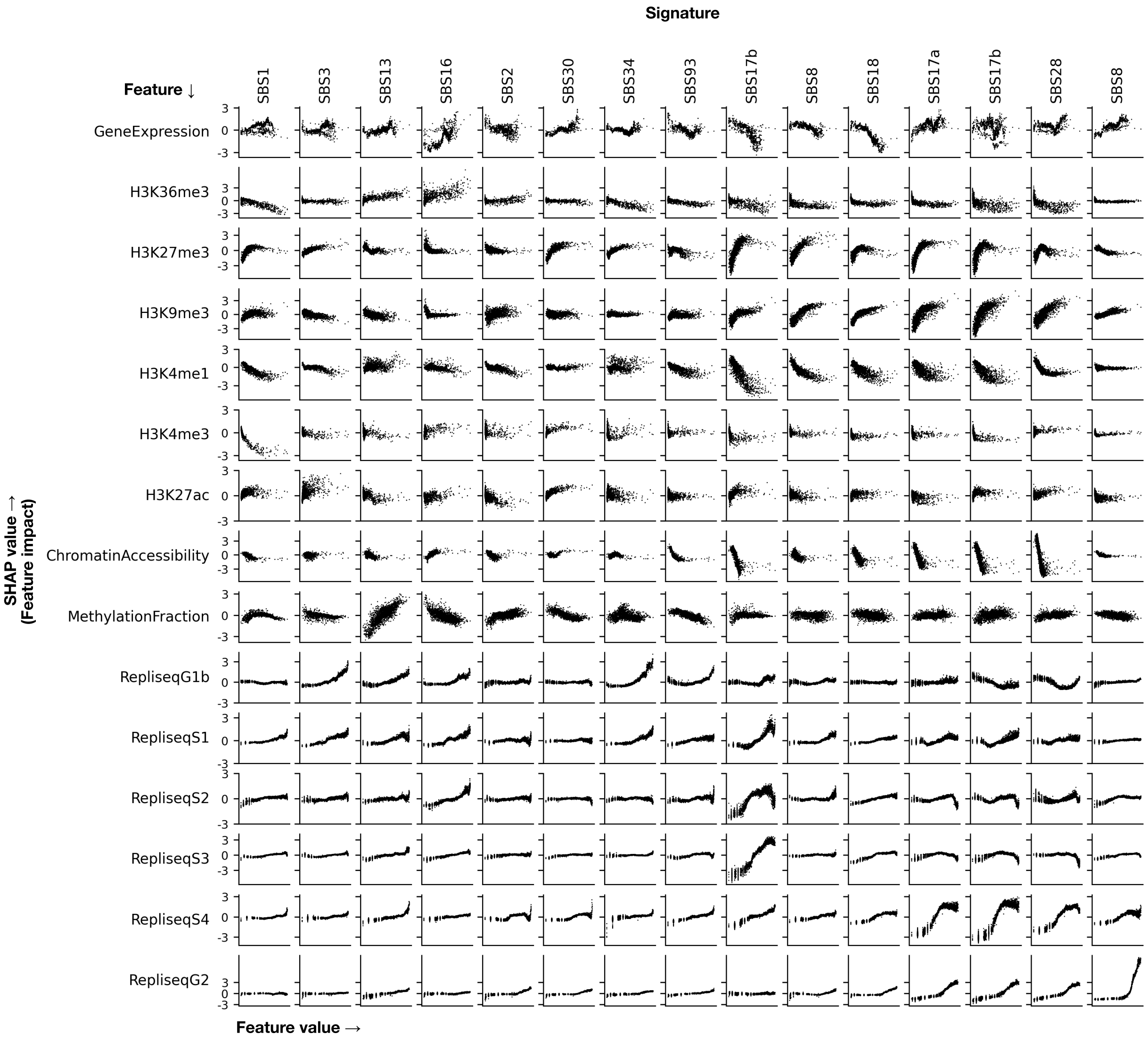

Supplementary Figure 9: Stomach adenocarcinoma Shapley value analysis per process, per genomic feature. Shapley values inferred for each signature and feature combination in the stomach adenocarcinoma dataset. Each point is a genomic region, which is assigned a feature value and feature impact for each signature.

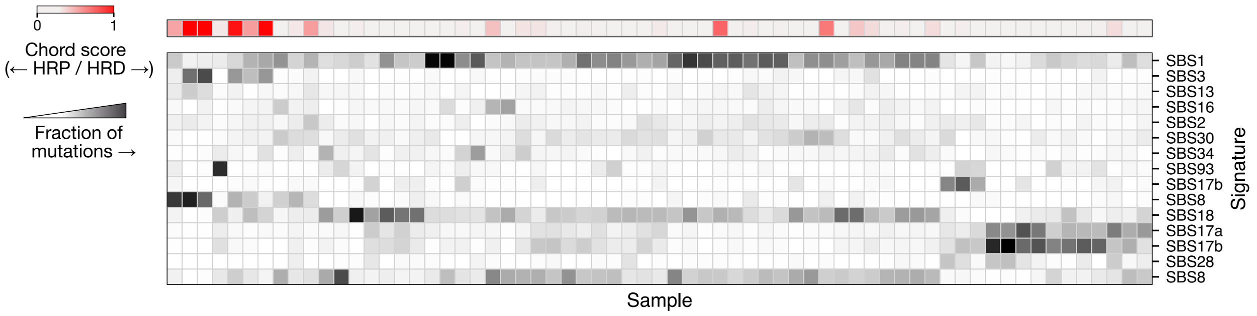

Supplementary Figure 10: Relative contributions per process in stomach adenocarcinoma samples. (top) CHORD HRD likelihood for each stomach adenocarcinoma sample. (bottom) Fraction of mutations assigned to each component.

bioRxiv preprint doi: https://doi.org/10.64898/2026.04.18.719374; this version posted April 21, 2026. The copyright holder for this preprint (which was not certified by peer review) is the author/funder, who has granted bioRxiv a license to display the preprint in perpetuity. It is made available under aCC-BY 4.0 International license.

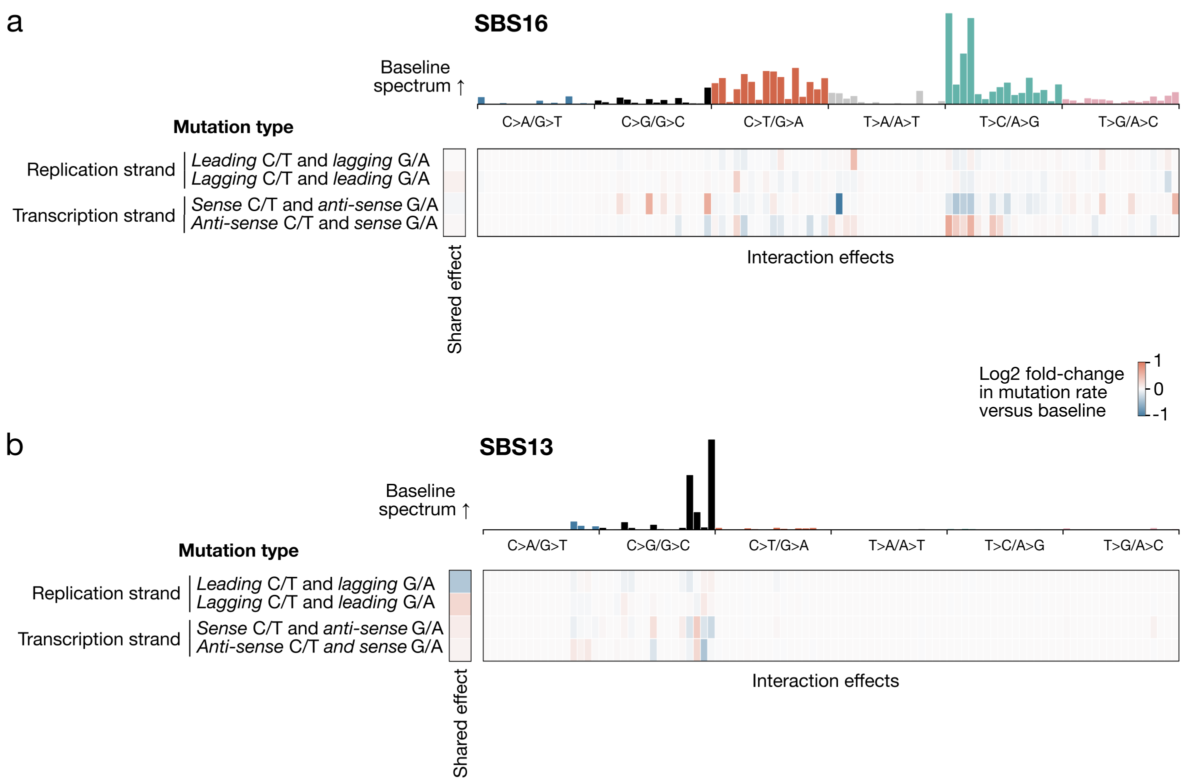

Supplementary Figure 11: Context interaction maps. Context interaction maps for signatures SBS16 (**a**) and SBS13 (**b**) from the stomach adenocarcinoma dataset.

bioRxiv preprint doi: https://doi.org/10.64898/2026.04.18.719374; this version posted April 21, 2026. The copyright holder for this preprint (which was not certified by peer review) is the author/funder, who has granted bioRxiv a license to display the preprint in perpetuity. It is made available under aCC-BY 4.0 International license.

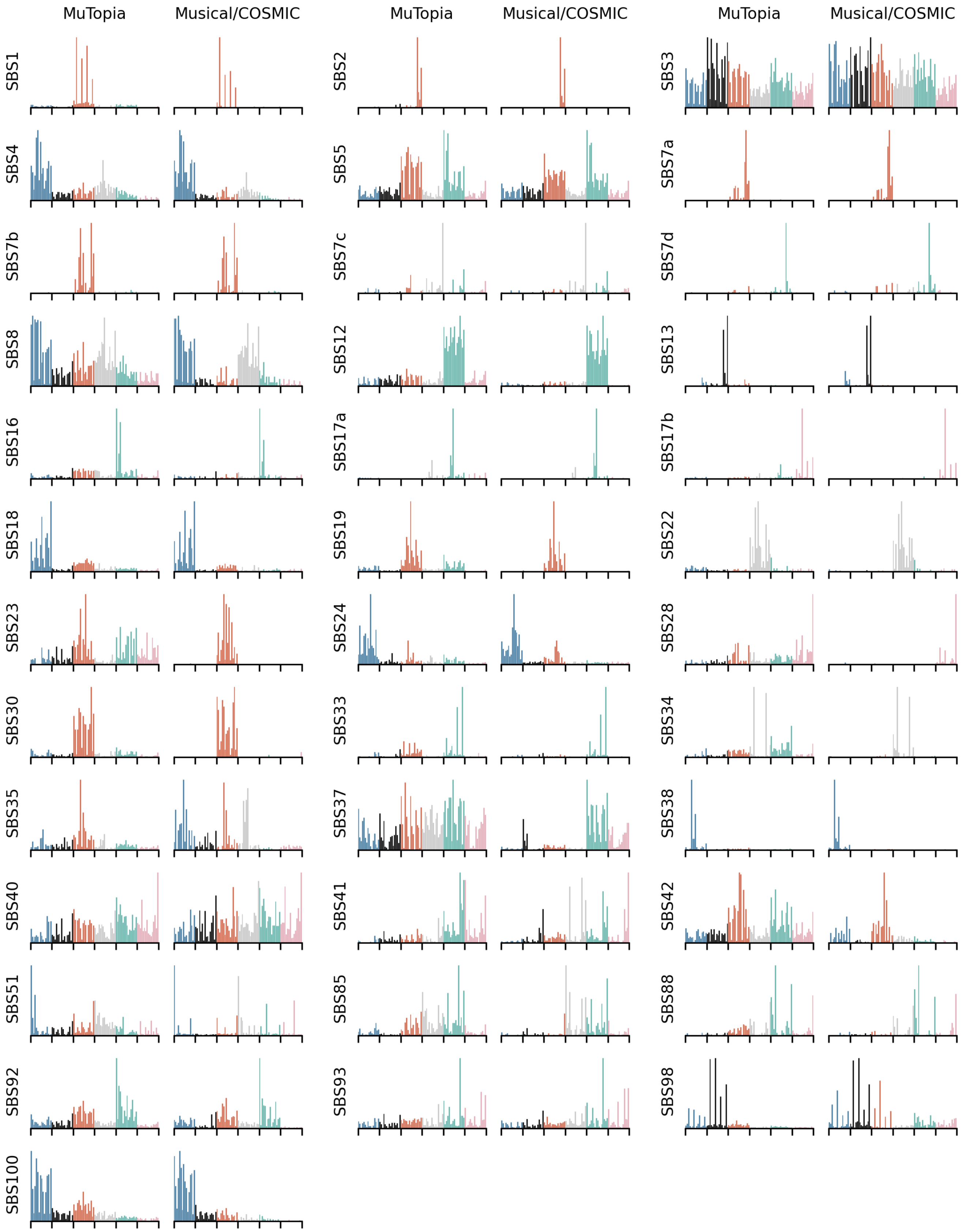

Supplementary Figure 12: COSMIC-matching signature spectra. The average mutational spectrum across all MuTopia components assigned to a COSMIC signature versus the MuSiCal/COSMIC representation.

bioRxiv preprint doi: https://doi.org/10.64898/2026.04.18.719374; this version posted April 21, 2026. The copyright holder for this preprint (which was not certified by peer review) is the author/funder, who has granted bioRxiv a license to display the preprint in perpetuity. It is made available under aCC-BY 4.0 International license.

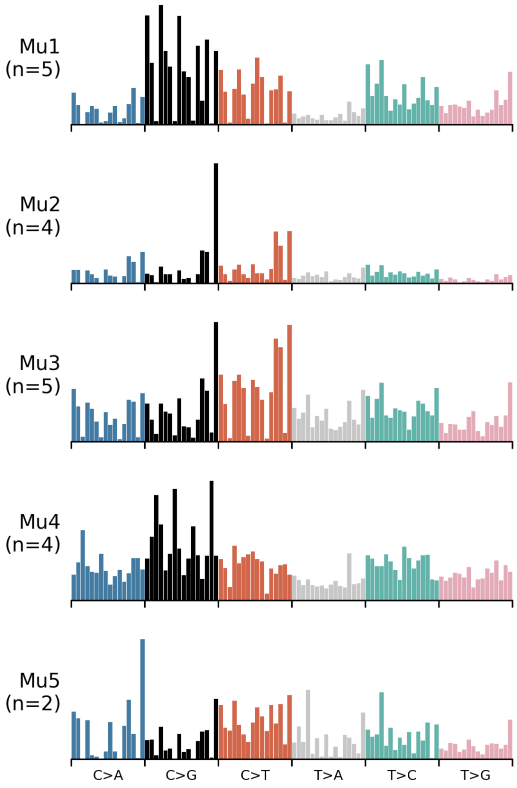

Supplementary Figure 13: Novel mutational signature spectra found in pan-cancer analysis. Novel mutational signatures recurrently discovered by MuTopia in multi-cancer meta-analysis, where “n” denotes the number of tumor types in which each process was found.

bioRxiv preprint doi: https://doi.org/10.64898/2026.04.18.719374; this version posted April 21, 2026. The copyright holder for this preprint (which was not certified by peer review) is the author/funder, who has granted bioRxiv a license to display the preprint in perpetuity. It is made available under aCC-BY 4.0 International license.

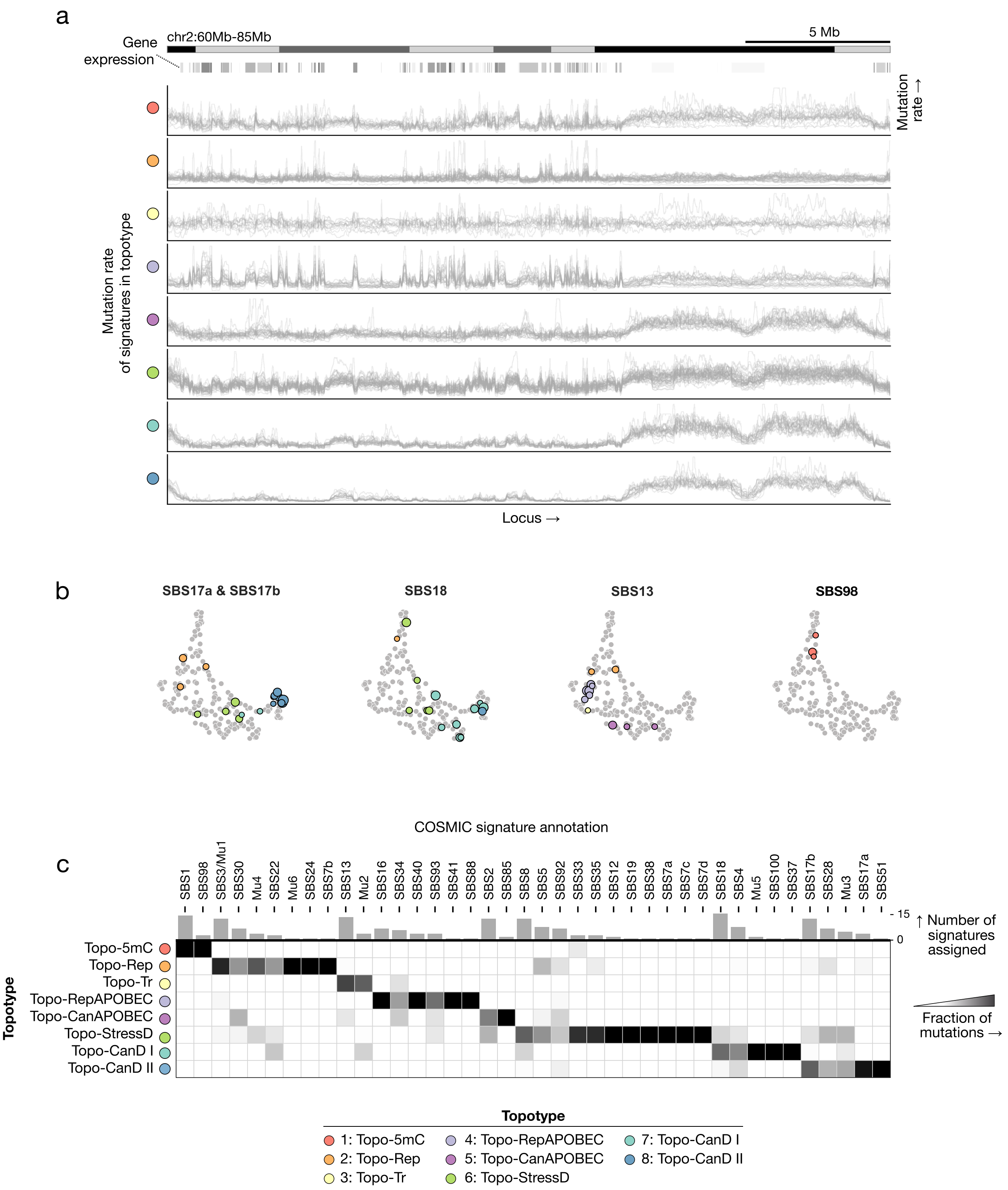

Supplementary Figure 14: Signature topotype assignments. **a)** Mutation rate profile for each signature for each topotype. **b)** Fraction of mutations attributed to signatures of each topotype, grouped by those signatures' COSMIC annotation. **c)** Signatures corresponding to COSMIC SBS annotations overlaid on the UMAP. For the points representing the highlighted signatures, the marker size indicates the log-transformed number of mutations.

bioRxiv preprint doi: https://doi.org/10.64898/2026.04.18.719374; this version posted April 21, 2026. The copyright holder for this preprint (which was not certified by peer review) is the author/funder, who has granted bioRxiv a license to display the preprint in perpetuity. It is made available under aCC-BY 4.0 International license.

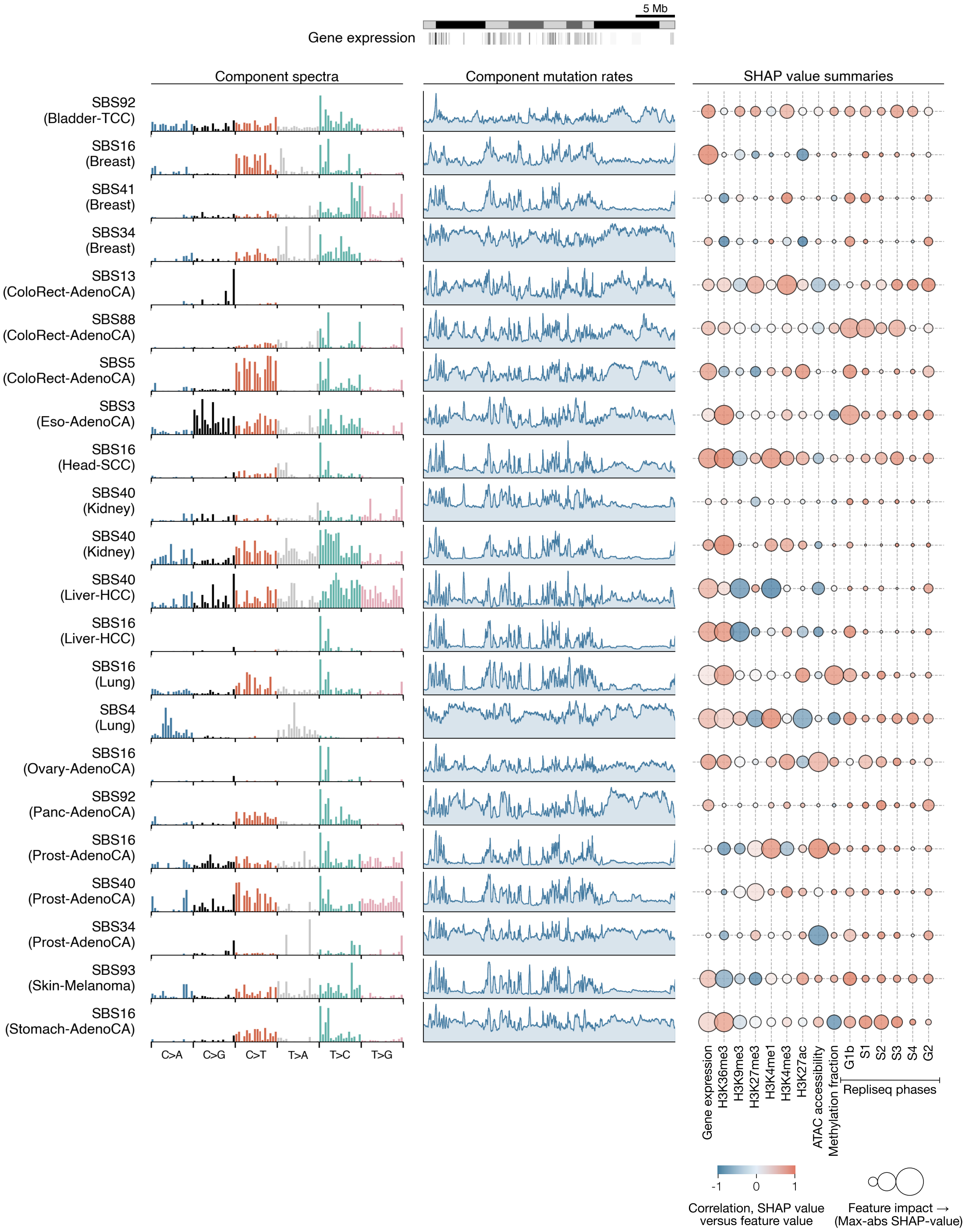

Supplementary Figure 15: Topo-Tr summary. Mutational spectra, mutation rate profiles, and Shapley value analysis of all signatures in topotype Topo-Tr (transcription).

bioRxiv preprint doi: https://doi.org/10.64898/2026.04.18.719374; this version posted April 21, 2026. The copyright holder for this preprint (which was not certified by peer review) is the author/funder, who has granted bioRxiv a license to display the preprint in perpetuity. It is made available under aCC-BY 4.0 International license.

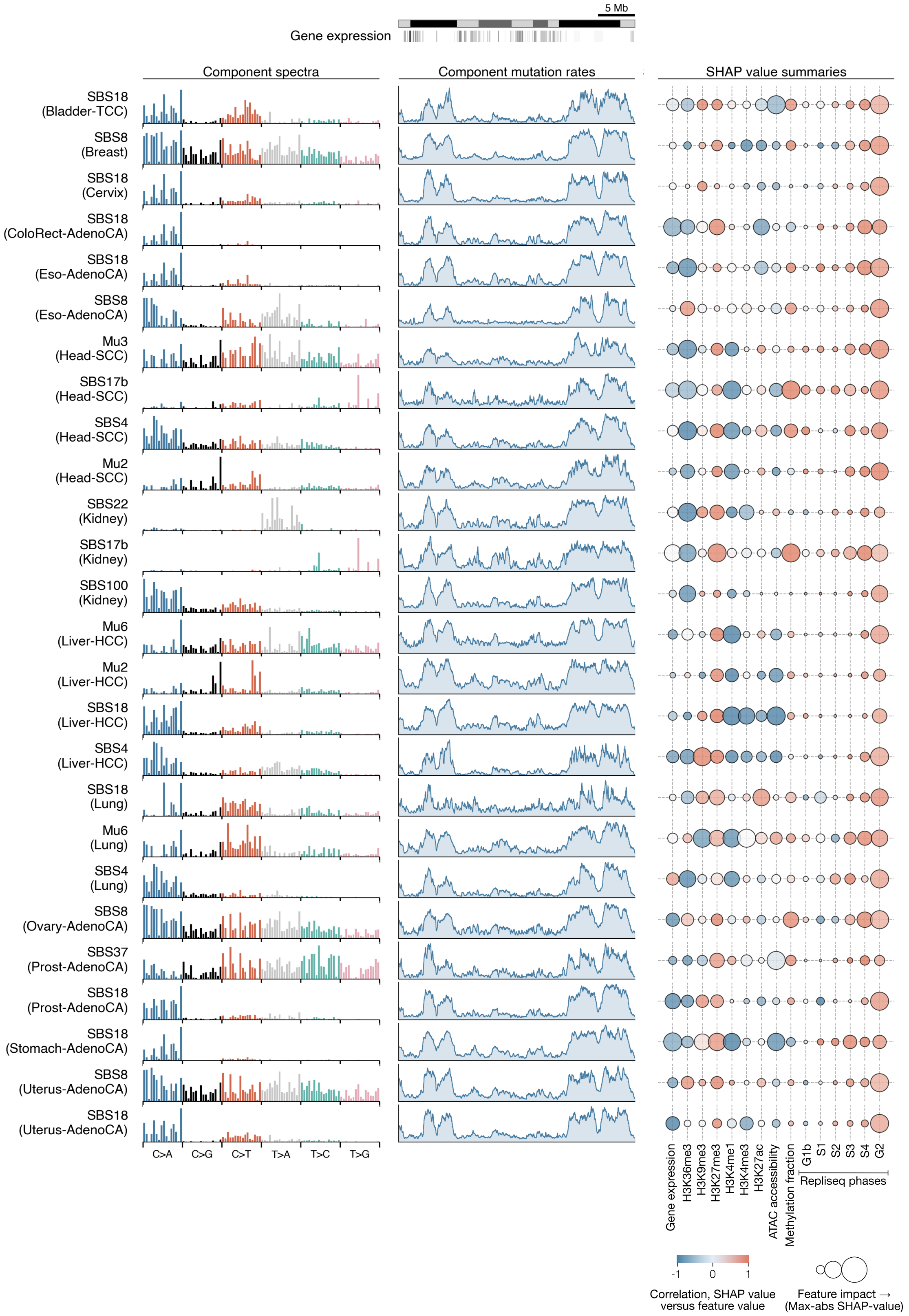

Supplementary Figure 16: Topo-CanD-I summary. Mutational spectra, mutation rate profiles, and Shapley value analysis of all signatures in topotype Topo-CanD-I (canonical damage I).

bioRxiv preprint doi: https://doi.org/10.64898/2026.04.18.719374; this version posted April 21, 2026. The copyright holder for this preprint (which was not certified by peer review) is the author/funder, who has granted bioRxiv a license to display the preprint in perpetuity. It is made available under aCC-BY 4.0 International license.

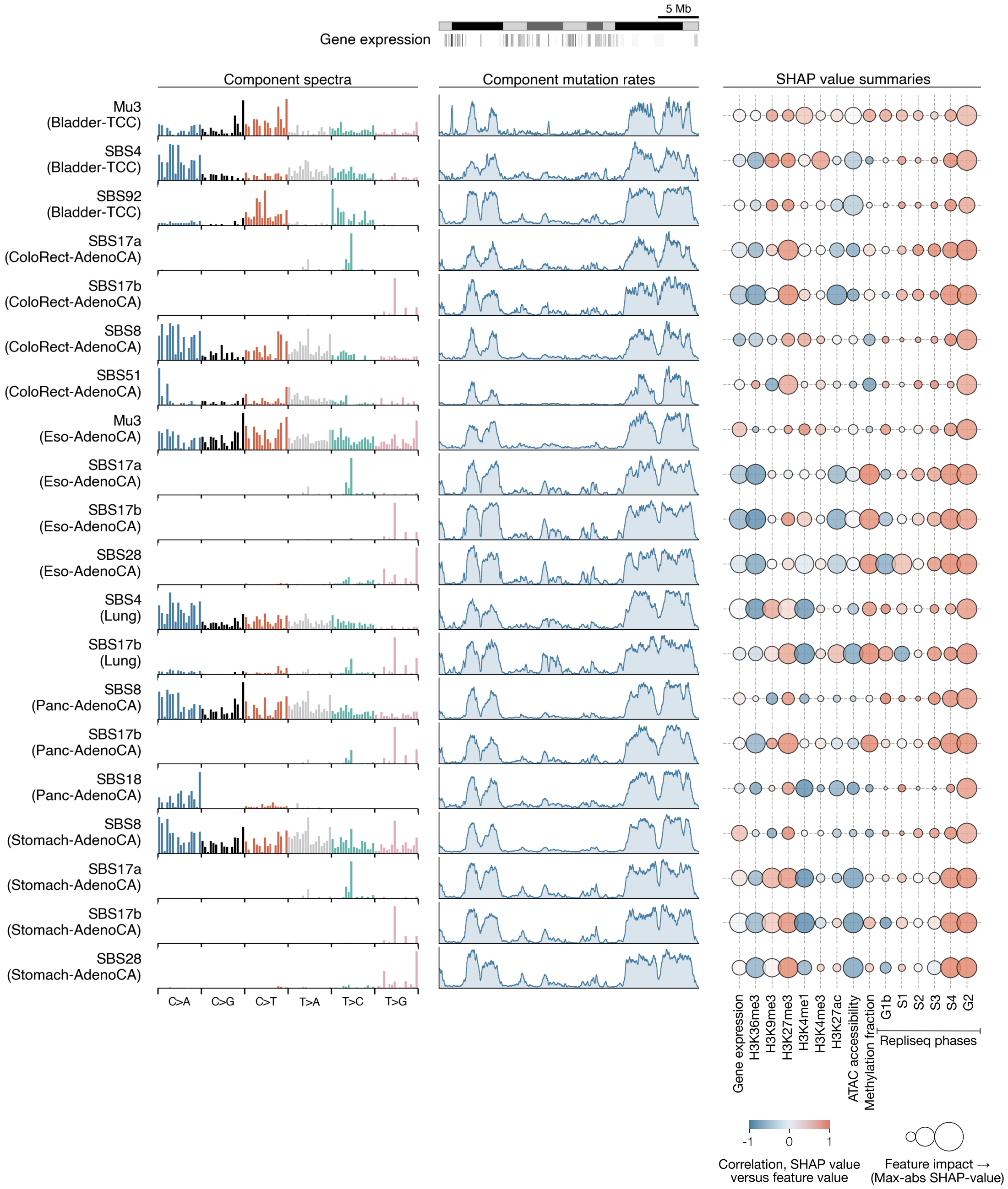

Supplementary Figure 17: Topo-CanD-II summary. Mutational spectra, mutation rate profiles, and Shapley value analysis of all signatures in topotype Topo-CanD-II (canonical damage II).

bioRxiv preprint doi: https://doi.org/10.64898/2026.04.18.719374; this version posted April 21, 2026. The copyright holder for this preprint (which was not certified by peer review) is the author/funder, who has granted bioRxiv a license to display the preprint in perpetuity. It is made available under aCC-BY 4.0 International license.

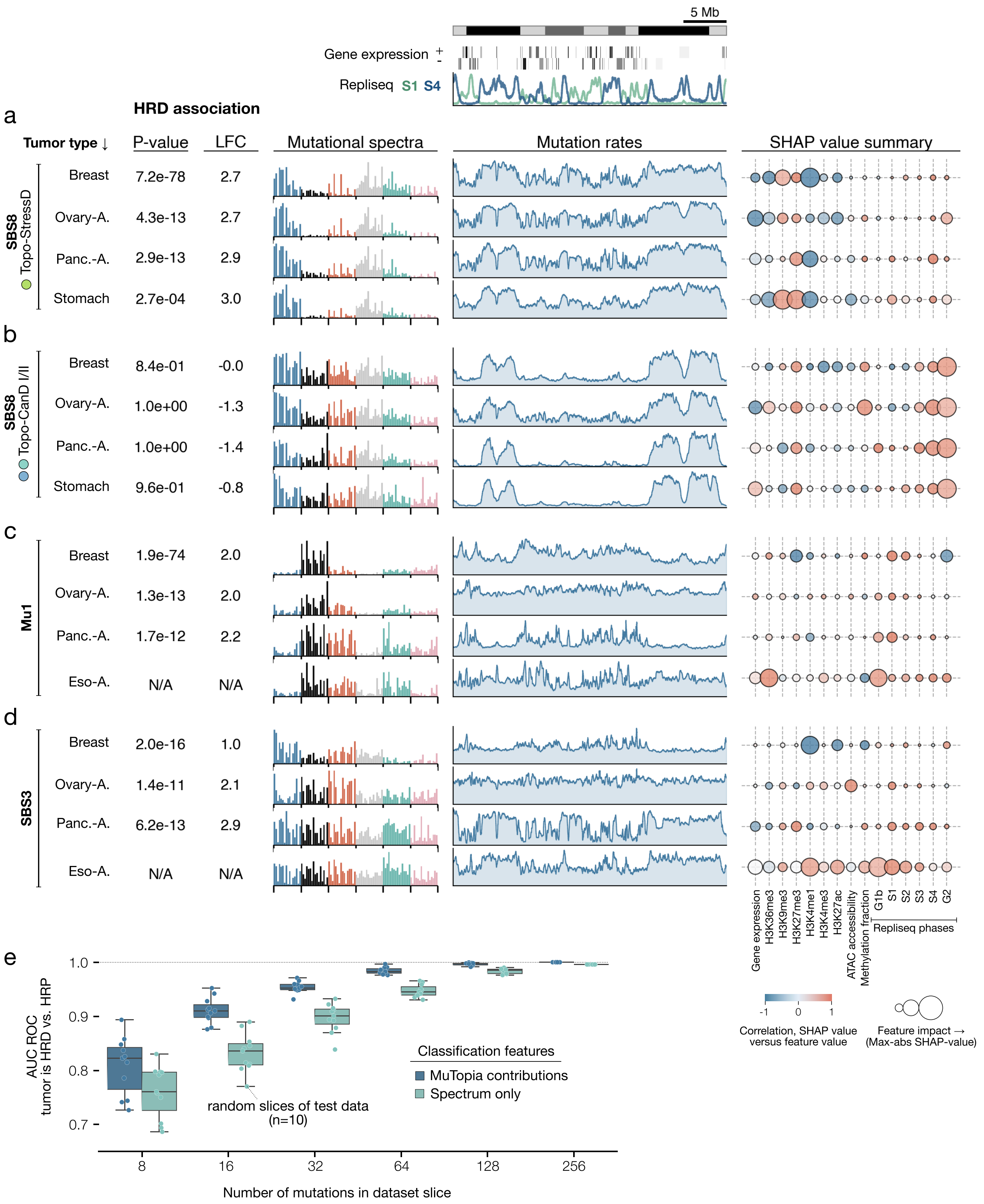

Supplementary Figure 18: HRD shapes mutational topography. a) Mutational spectra, predicted mutation rate profiles, and Shapley value analysis for SBS8 signatures in the Topo-StressD topotype. The p-value and LFC (log2 fold-change) statistics show the enrichment of each process in HRD versus HRP samples, as predicted by CHORD. (Mann–Whitney U-test, Benjamini–Hochberg correction.) **b)** Same as in (a), but for SBS8 signatures in the Topo-CanD-I/II topotypes in the same tumor types. **c)** Same as in (a), but for Mu1 signatures. No esophageal adenocarcinoma samples were called HRD-positive, so these were labeled “N/A”. **d)** Same as in (c), but for SBS3 signatures in the same tumor types. **e)** HRD classification performance of a logistic regression model trained on either the mutational spectra of ovarian adenocarcinoma whole genome samples, or the contributions of each mutational process to the same tumors as inferred by the MuTopia model. We sub-sampled the test-set samples to assess the minimum number of mutations needed for classification using both feature sets.

bioRxiv preprint doi: https://doi.org/10.64898/2026.04.18.719374; this version posted April 21, 2026. The copyright holder for this preprint (which was not certified by peer review) is the author/funder, who has granted bioRxiv a license to display the preprint in perpetuity. It is made available under aCC-BY 4.0 International license.

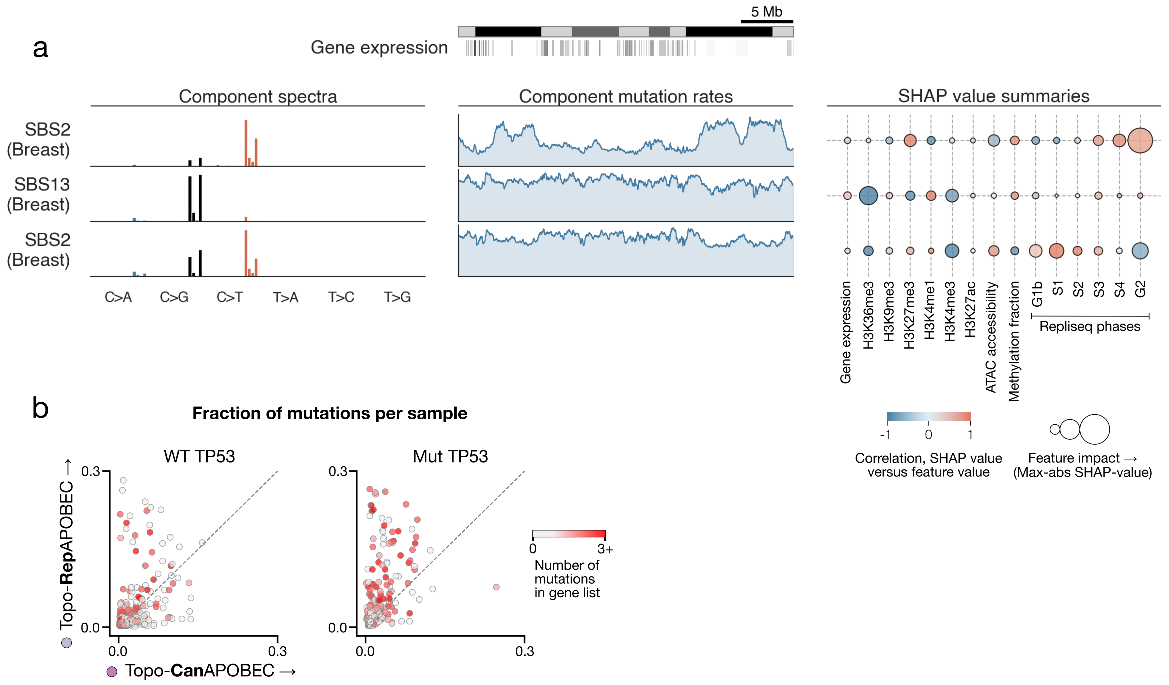

Supplementary Figure 19: APOBEC exhibits varied genomic mutational profiles. a) Mutational spectra, mutation rate profiles, and Shapley value analysis for APOBEC signatures extracted from the breast tumor dataset. **b)** Fraction of mutations per sample attributed to Topo-CanAPOBEC versus Topo-RepAPOBEC, stratified by TP53 mutation status and colored by number of mutations in cancer driver genes.

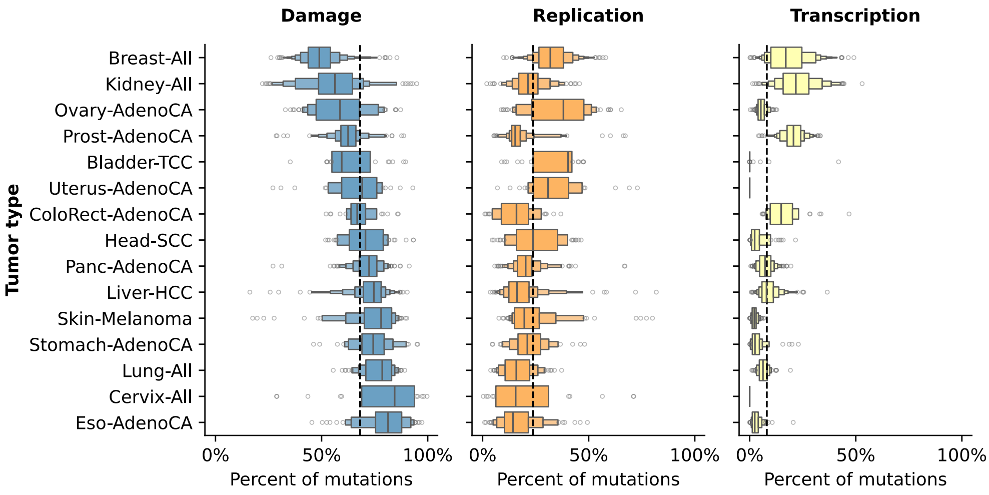

Supplementary Figure 20: Fractions of damage, replication, and transcription-associated mutagenesis. Relative fraction of mutations from damage topotypes (Topo-CanD-I/II, Topo-CanAPOBEC, Topo-StressD), replication topotypes (Topo-Rep, Topo-RepAPOBEC), and transcription topotypes (Topo-Tr) across samples for each tumor type. The dashed line shows the average across tumor types.

bioRxiv preprint doi: https://doi.org/10.64898/2026.04.18.719374; this version posted April 21, 2026. The copyright holder for this preprint (which was not certified by peer review) is the author/funder, who has granted bioRxiv a license to display the preprint in perpetuity. It is made available under aCC-BY 4.0 International license.

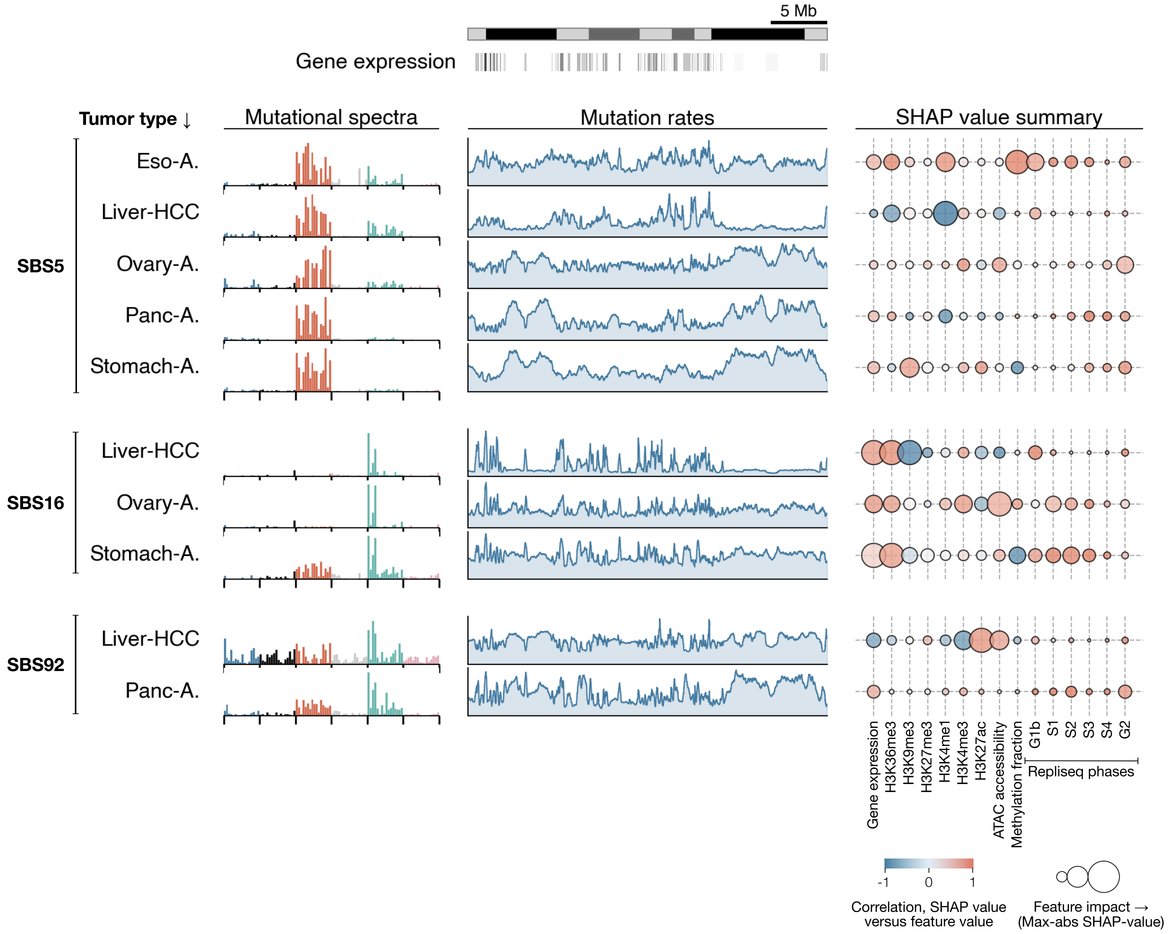

Supplementary Figure 21: SBS5 as a composite signature of SBS30, SBS16, and SBS92. Mutational spectra, mutation rate profiles, and Shapley value analysis of SBS30-, SBS92-, and SBS16-designated signatures from tumor types which did not contain SBS5.
